# Efficacy of antibiotic prophylaxis in patients with cancer and hematopoietic stem cell transplantation recipients: A systematic review of randomized trials

**DOI:** 10.1002/cam4.2395

**Published:** 2019-07-05

**Authors:** Grace Egan, Paula D. Robinson, Juan P. D. Martinez, Sarah Alexander, Roland A. Ammann, L. Lee Dupuis, Brian T. Fisher, Thomas Lehrnbecher, Bob Phillips, Sandra Cabral, George Tomlinson, Lillian Sung

**Affiliations:** ^1^ Division of Haematology/Oncology The Hospital for Sick Children Toronto Ontario Canada; ^2^ Pediatric Oncology Group of Ontario Toronto Ontario Canada; ^3^ Biostatistics Research Unit Toronto General Hospital Toronto Ontario Canada; ^4^ Division of Pediatric Hematology/Oncology, Department of Pediatrics, Inselspital Bern University Hospital, University of Bern Bern Switzerland; ^5^ Department of Pharmacy and Research Institute, The Hospital for Sick Children, and Leslie Dan Faculty of Pharmacy University of Toronto, The Hospital for Sick Children Toronto Ontario Canada; ^6^ Division of Infectious Diseases Children's Hospital of Philadelphia Philadelphia PA USA; ^7^ Pediatric Hematology and Oncology Johann Wolfgang Goethe University Frankfurt Germany; ^8^ Leeds Children's Hospital, Leeds General Infirmary, Leeds Teaching Hospitals, NHS Trust, Leeds, United Kingdom and Centre for Reviews and Dissemination, University of York Leeds West Yorkshire UK; ^9^ Institute of Health Policy Management and Evaluation University of Toronto Toronto Ontario Canada

**Keywords:** antibiotic prophylaxis, cancer, meta‐analysis, randomized trials

## Abstract

**Purpose:**

To determine the efficacy and safety of different prophylactic systemic antibiotics in adult and pediatric patients receiving chemotherapy or undergoing hematopoietic stem cell transplantation (HSCT).

**Methods:**

We conducted a systematic review and performed searches of Ovid MEDLINE, MEDLINE in‐process and Embase; and Cochrane Central Register of Controlled Trials. Studies were included if patients had cancer or were HSCT recipients with anticipated neutropenia, and the intervention was systemic antibacterial prophylaxis. Strategies synthesized included fluoroquinolone vs no antibiotic/nonabsorbable antibiotic; fluoroquinolone vs trimethoprim‐sulfamethoxazole; trimethoprim‐sulfamethoxazole vs no antibiotic; and cephalosporin vs. no antibiotic. Fluoroquinolone vs cephalosporin and levofloxacin vs ciprofloxacin were compared by network meta‐analysis. Primary outcome was bacteremia.

**Results:**

Of 20 984 citations screened, 113 studies comparing prophylactic antibiotic to control were included. The following were effective in reducing bacteremia: fluoroquinolone vs no antibiotic/nonabsorbable antibiotic (risk ratio (RR) 0.56, 95% confidence interval (CI) 0.41‐0.76), trimethoprim‐sulfamethoxazole vs no antibiotic (RR 0.59, 95% CI 0.41‐0.85) and cephalosporin vs no antibiotic (RR 0.30, 95% CI 0.16‐0.58). Fluoroquinolone was not significantly associated with increased *Clostridium difficile* infection (RR 0.62, 95% CI 0.31‐1.24) or invasive fungal disease (RR 1.28, 95% CI 0.79‐2.08) but did increase resistance to fluoroquinolone among bacteremia isolates (RR 3.35, 95% CI 1.12 to 10.03). Heterogeneity in fluoroquinolone effect on bacteremia was not explained by evaluated study, population, or methodological factors. Network meta‐analysis revealed no direct comparisons for pre‐specified analyses; superior regimens were not identified.

**Conclusions:**

Fluoroquinolone, trimethoprim‐sulfamethoxazole, and cephalosporin prophylaxis reduced bacteremia. A clinical practice guideline to facilitate prophylactic antibiotic decision‐making is required.

## 1 INTRODUCTION

Bacteremia and infectious complications are important causes of morbidity and death in children and adults receiving intensive chemotherapy and undergoing hematopoietic stem cell transplant (HSCT).^1^, ^2^ A number of preventative strategies to reduce infection in neutropenic patients have been investigated, including granulocyte infusions,^3^ granulocyte colony‐stimulating factor (G‐CSF),^4^, ^5^ nonabsorbable antibiotics,^6^ and systemic antibiotics.^7^ The potential efficacy of systemically administered antibiotic prophylaxis is of great interest.^7^ However, there is uncertainty regarding the optimal prophylactic antibiotic class in terms of efficacy and adverse effects. Outcomes important in this decision include measures of prophylaxis efficacy including bacteremia, fever, and mortality. In addition, the evaluation of potential adverse effects should be considered including antibiotic resistance, *Clostridium difficile* infection, and invasive fungal disease. Also, adverse effects associated with specific antibiotic classes such as fluoroquinolone‐related musculoskeletal toxicities warrant consideration.

There are many randomized trials that have evaluated systemic antibiotic prophylaxis in patients with cancer and HSCT recipients. Furthermore, the Children's Oncology Group recently published a large randomized trial of 624 high‐risk pediatric patients evaluating levofloxacin prophylaxis, thus substantially increasing the pediatric evidence base around antibiotic prophylaxis.^8^ Therefore, we reasoned it would be timely to perform a systematic review of antibiotic prophylaxis in order to inform a future evidence‐based clinical practice guideline. Also, since treatment‐related mortality is declining over time,^9^ the impact of prophylaxis on mortality may be changing, thus increasing the importance of conducting the analysis with recently conducted studies.

Consequently, our objectives were to determine the efficacy and safety of different prophylactic systemic antibiotics in patients receiving chemotherapy or undergoing HSCT.

## 2 METHODS

For this systematic review, we followed the Preferred Reporting Items for Systematic Reviews and Meta‐Analyses (PRISMA) recommendations for reporting.^10^

### 2.1 Data sources and searches

With the assistance of a library scientist, we searched Ovid MEDLINE, MEDLINE in‐process and Embase; and Wiley Cochrane Central Register of Controlled Trials for articles indexed up to 26 November 2018. The search strategy included the Medical Subject Heading terms and text words that identified patients with cancer or HSCT recipients receiving antibacterial prophylaxis (Supplemental Appendix S1 contains the full search strategy). The resultant set was limited to randomized trials published in 1980 or later. There was no restriction by language.

### 2.2 Study selection

We defined inclusion and exclusion criteria a priori. Studies were included if the manuscript was a fully published primary randomized or quasi‐randomized trial with a parallel group design; if the study compared the administration of a systemic antibacterial agent to any control group as prophylaxis; and if at least 90% of participants were patients undergoing chemotherapy for cancer or HSCT for any indication. As trimethoprim‐sulfamethoxazole can be administered as both prophylaxis against bacterial infection (daily) and *Pneumocystis jiroveci* pneumonia (intermittently), systemic antibiotic prophylaxis with trimethoprim‐sulfamethoxazole required administration at least once daily. Reasons for excluding studies were as follows: (a) not a full text publication; (b) not a randomized trial with a parallel group design; (c) less than 90% patients receiving chemotherapy for cancer or undergoing HSCT; (d) intervention not a systemic antibacterial agent administered for prophylaxis; (e) antibacterial agent given as peri‐procedural prophylaxis only; (e) duplicate study; and (f) published before 1980.

Two reviewers (GE and PDR) independently evaluated the titles and abstracts of publications identified by the search strategy and all potentially relevant publications were retrieved in full. Disagreements between the two reviewers were resolved by consensus and adjudicated by a third reviewer (LS) if required. We described agreement with study inclusion between the two reviewers using the kappa statistic and agreement was defined as slight (0 to 20%), fair (21 to 40%), moderate (41 to 60%), substantial (61 to 80%) or almost perfect (81 to 100%).^11^

### 2.3 Data abstraction and methodological approach

Two reviewers (GE and PDR) abstracted all data in duplicate and any discrepancies were resolved by consensus. A third reviewer (LS) resolved any outstanding discrepancies if required. Efficacy outcome measures were bacteremia, fever, neutropenic fever, infection‐related mortality, and overall mortality. Episodes of fever and neutropenic fever were abstracted into different categories as some studies described any fever irrespective of neutrophil count while other studies only described fever if it occurred during neutropenia. Adverse outcome measures were *C difficile* infection, invasive fungal disease (as defined by each study), musculoskeletal adverse effects and antibiotic resistance. Antibiotic resistance to the intervention antibiotic among all bacteremia isolates tested was abstracted from intervention and control groups. This outcome was not evaluated in studies comparing two systemic prophylactic antibiotics.

Study‐level factors collected included year of study publication, number of randomized groups, country of study conduct, age group (adult, pediatric or both), age range, treatment group (cancer patients receiving chemotherapy only, HSCT only, or both chemotherapy and HSCT) and cancer diagnosis or HSCT type. Pediatric studies were defined as those in which all participants were less than 25 years of age while adult studies were defined as those in which all participants were older than 15 years of age.

### 2.4 Interventions evaluated

Based upon the available data and clinical relevance, comparisons at the group level focused broadly on fluoroquinolone‐based and non‐fluoroquinolone‐based evaluations. The fluoroquinolone analysis concentrated on the comparison between fluoroquinolone vs no antibiotic or nonabsorbable antibiotic. These control groups were combined as we presumed that nonabsorbable antibiotic would have minimal impact on the efficacy and safety outcomes of interest, and combining them would improve power to identify sources of heterogeneity. However, we also presented the analysis stratified by no antibiotic and nonabsorbable antibiotic control groups separately. No antibiotic controls included both placebo and usual care (no antibiotic prophylaxis). Next, we evaluated the impact of levofloxacin and ciprofloxacin specifically vs no antibiotic. Finally, we compared fluoroquinolone vs trimethoprim‐sulfamethoxazole prophylaxis.

In terms of non‐fluoroquinolone‐based comparisons, we compared trimethoprim‐sulfamethoxazole vs no antibiotic, cephalosporin vs no antibiotic, parenteral glycopeptide vs no antibiotic and rifampin plus fluoroquinolone vs fluoroquinolone.

For trials with more than two study arms the following hierarchical rules were used to determine the intervention and control groups for conventional meta‐analysis although all arms were used for network meta‐analysis. Control group was chosen in the following order: (a) placebo; (b) no antibiotic; and (c) nonabsorbable antibiotic. If different fluoroquinolones were examined, the fluoroquinolone with the broadest spectrum of activity was considered the intervention.

### 2.5 Assessment of risk of bias

Two reviewers (GE and PDR) assessed study quality and any discrepancies were resolved by consensus. Outstanding discrepancies were resolved by a third reviewer (LS) if required. Study quality was evaluated at the level of the study using the Cochrane Collaboration's tool for assessing the risk of bias in randomized trials.^12^ It includes the following domains relevant to internal validity: selection bias, performance bias, detection bias, attrition bias, and reporting bias. We evaluated the following sources of bias related to these domains: random number generation, allocation concealment, blinding of participants and personnel, blinding of outcome assessment, incomplete outcome data, and selective outcome reporting.

### 2.6 Statistical methods

We combined data at the study level for this meta‐analysis. Synthesis was conducted when there were at least three studies that reported an outcome for a main comparison and at least two studies that reported an outcome within each stratum in the stratified analysis. Data were synthesized using the risk ratio (RR) as the effect measure with its 95% confidence interval (CI). The Mantel‐Haenszel approach was used to estimate treat effects and effects were weighted by the inverse variance. In this analysis, RR < 1 suggests that the intervention is better than the control group. As we anticipated heterogeneity between studies, a random effects model (DerSimonian and Laird)^13^ was used for all analyses. Statistical heterogeneity between trials was assessed using the I^2^ value, which describes the percentage of total variation across studies due to heterogeneity rather than chance.^12^

For stratified analysis, we a priori prioritized evaluation of fluoroquinolone based on the large number of available trials, broad Gram‐negative coverage and fewer myelosuppression concerns.^7^ We evaluated the following factors to identify if they could explain heterogeneity in prophylaxis effect: treatment group (chemotherapy, HSCT or both), age of participants (adult or pediatric), year of publication (<2000 vs ≥2000), risk of bacteremia in control arm (< median among the whole cohort vs ≥ median), adequate sequence generation, and adequate allocation concealment. We determined if the effect varied by subgroup through evaluation of the P value for interaction. Only stratified analyses for the primary outcome of bacteremia and the key secondary outcome of overall survival were conducted to limit the number of tests performed.

For network meta‐analysis, we focused on comparisons of interest in which direct comparison was limited or not available because of the paucity/absence of head‐to‐head trials. These included comparison of fluoroquinolone vs cephalosporin and levofloxacin vs ciprofloxacin. Only studies that assessed any of these antibiotic types were included in the network. Network meta‐analysis was restricted to the primary outcome of bacteremia.

Potential publication bias was explored by visual inspection of funnel plots when at least 10 studies were available.^12^ Funnel plots graphically display the effect measure on the X‐axis and precision on the Y‐axis. Asymmetry with an absence of studies in a lower quadrant may indicate publication bias. In the event of such asymmetry, we used the trim and fill technique to describe the potential impact of such bias. With this approach, outlying studies are removed and hypothetical negative studies with equal weight are added.^12^

We synthesized data for conventional meta‐analysis using Review Manager 5.3 (Cochrane Collaboration, Nordic Cochrane Centre). Network meta‐analysis was conducted using a Bayesian approach using R through the library gemtc.

## 3 RESULTS

**Figure 1:**
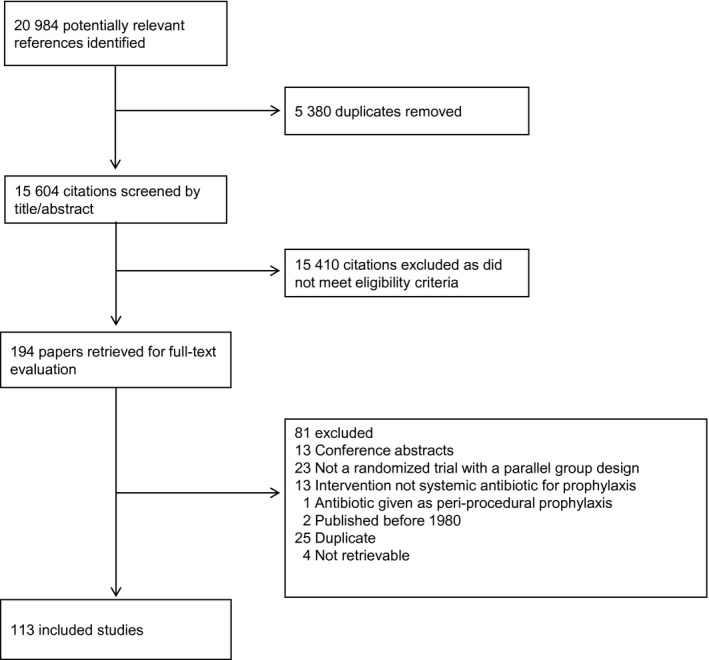
Flow diagram depicting study identification, selection, and reasons for exclusion

The flow of study identification and selection is illustrated in Figure 1. There were 20,984 citations identified by the search strategy, of which 194 were retrieved for full‐text evaluation. Of these papers, 113 met the eligibility criteria and were included in the systematic review. Figure 1 describes reasons for exclusion. Agreement in study inclusion between the two reviewers was almost perfect with kappa = 97.9% (95% CI 95.0‐100).

Table 1 describes the characteristics of the 113 included studies with 13,677 patients; details are shown in Supplemental Appendix S2. There were 73 studies (65%) consisting of patients with cancer receiving chemotherapy, 19 (17%) consisting of patients undergoing HSCT and 21 (18%) consisting of both chemotherapy and HSCT recipients. Only 13 (12%) studies were solely pediatric. Trials were conducted in 20 different countries. The most common antibiotic comparison available for synthesis was fluoroquinolone vs no antibiotic or nonabsorbable antibiotic (n = 29), which were divided into no antibiotic (n = 24) and nonabsorbable antibiotic (n = 5) control groups. The second most common antibiotic comparison available for synthesis was trimethoprim‐sulfamethoxazole vs no antibiotic (n = 18).

**Table 1:** Characteristics of included studies in systematic review (N = 113)

Characteristic and strata	No. studies (%)
Study population characteristics	
Treatment	
Cancer patients receiving chemotherapy only	73 (65%)
Hematopoietic stem cell transplantation only	19 (17%)
Both chemotherapy and transplantation	21 (18%)
Age participants	
Adult	75 (66%)
Pediatric	13 (12%)
Both	18 (16%)
Not stated	7 (6%)
Interventions included in synthesis^a^	
Fluoroquinolone vs no antibiotic or nonabsorbable antibiotic	29
Fluoroquinolone vs no antibiotic	24
Levofloxacin vs no antibiotic	5
Ciprofloxacin vs no antibiotic	5
Fluoroquinolone vs non‐absorbable antibiotic	5
Fluoroquinolone vs trimethoprim‐sulfamethoxazole	8
Trimethoprim‐sulfamethoxazole vs no antibiotic	18
Cephalosporin vs no antibiotic	4
Parenteral glycopeptide vs no antibiotic	4
Rifampin plus fluoroquinolone vs fluoroquinolone	3
Risk of bias	
Adequate sequence generation	24 (21%)
Adequate allocation concealment	21 (19%)
Participants and personnel blinded	27 (24%)
Outcome assessors blinded	11 (10%)
Lack of attrition bias	70 (62%)
Free of selective reporting	33 (29%)

a No antibiotic includes placebo and usual care (no antibiotic prophylaxis) control groups.

**Figure 2:**
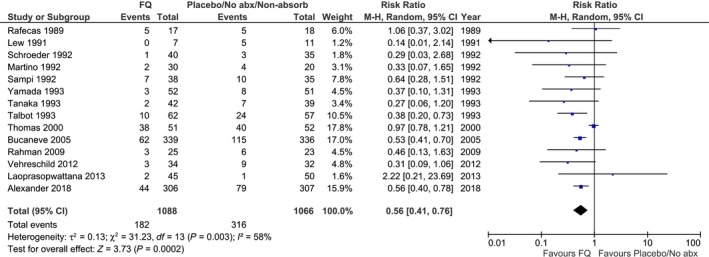
Forest plot of bacteremia rate among studies comparing any fluroquinolone vs no antibiotic or nonabsorbable antibiotic. Squares to the left of the vertical line mean that bacteremia was reduced with fluoroquinolone. Horizontal lines through the squares represent 95% confidence intervals (CIs). The size of the squares reflects each study's relative weight, and the diamond represents the aggregate risk ratio and 95% CI

Table 2 shows the synthesized outcomes for fluoroquinolone‐based comparisons. When compared to no antibiotic or nonabsorbable antibiotic controls, fluoroquinolone significantly reduced bacteremia (RR 0.56, 95% CI 0.41‐0.76) (Figure 2), fever (RR 0.78, 95% CI 0.66‐0.93), neutropenic fever (RR 0.87, 95% CI 0.82‐0.93) and infection‐related mortality (RR 0.64, 95% CI 0.42‐0.98) but did not significantly reduce overall mortality (RR 0.85, 95% CI 0.65‐1.11). Fluoroquinolone prophylaxis was not associated with a significant increase in *C difficile* infection, invasive fungal disease or musculoskeletal toxicity. However, fluoroquinolone resistance was increased among bacteremia isolates in the prophylaxis group (RR 3.35, 95% CI 1.12‐10.03). These results were almost identical to comparison of fluoroquinolone vs. no antibiotic control separately. In evaluating specific fluoroquinolones, levofloxacin significantly reduced bacteremia (RR 0.54, 95% CI 0.44‐0.67), fever (RR 0.63, 95% CI 0.42‐0.95) and neutropenic fever (RR 0.87, 95% CI 0.80‐0.95) without significantly reducing overall mortality (RR 0.79, 95% CI 0.52‐1.20) while ciprofloxacin did not significantly reduce bacteremia, neutropenic fever or overall mortality compared to no antibiotic. To evaluate whether specific fluoroquinolone (levofloxacin, ciprofloxacin, norfloxacin, or ofloxacin) explained heterogeneity in the effect of fluoroquinolone prophylaxis vs no antibiotic to reduce bacteremia, the *P* value for interaction was 0.74. Comparison between fluoroquinolone and trimethoprim‐sulfamethoxazole did not show significant differences in bacteremia, fever, infection‐related mortality, or invasive fungal disease.

**Table 2:** Synthesized outcomes for comparisons of fluoroquinolone prophylaxis^a^

Comparison and outcomes	Number studies	Number patients	RR	95% CI	*I* ^2^	*P*
A. Fluoroquinolone vs No Antibiotic or Non‐absorbable Antibiotic Comparisons						
1. Fluoroquinolone vs either no antibiotic or non‐absorbable antibiotic						
Bacteremia	14	2154	0.56	0.41‐0.76	58%	0.0002
Fever	12	3231	0.78	0.66‐0.93	84%	0.005
Neutropenic fever	9	1302	0.87	0.82‐0.93	0%	< 0.0001
Infection‐related mortality	19	4376	0.64	0.42‐0.98	0%	0.04
Overall mortality	17	3742	0.85	0.65‐1.11	15%	0.24
*C difficile* infection	3	798	0.62	0.31‐1.24	0%	0.17
Invasive fungal disease	8	1242	1.28	0.79‐2.08	0%	0.31
Musculoskeletal adverse effects	3	1272	0.70	0.44‐1.12	0%	0.14
Antibiotic resistance	4	147	3.35	1.12‐10.03	64%	0.03
2. Fluoroquinolone vs no antibiotic						
a) All fluoroquinolone vs no antibiotic						
Bacteremia	14	2154	0.56	0.41‐0.76	58%	0.0002
Fever	9	2996	0.70	0.57‐0.86	71%	0.0008
Neutropenic fever	8	1174	0.88	0.82‐0.95	0%	0.0008
Infection‐related mortality	16	4016	0.72	0.45‐1.16	0%	0.17
Overall mortality	15	3444	0.86	0.62‐1.17	24%	0.34
*C difficile* infection	3	798	0.62	0.31‐1.24	0%	0.17
Invasive fungal disease	6	1032	1.25	0.75‐2.08	0%	0.39
Musculoskeletal adverse effects	3	1272	0.66	0.39‐1.13	0%	0.13
Antibiotic resistance	4	147	3.35	1.12‐10.03	64%	0.03
b) Levofloxacin vs no antibiotic						
Bacteremia	3	1336	0.54	0.44‐0.67	0%	<0.00001
Fever	3	2490	0.63	0.42‐0.95	73%	0.03
Neutropenic fever	3	880	0.87	0.80‐0.95	0%	0.002
Infection‐related mortality	4	3101	0.72	0.36‐1.43	0%	0.35
Overall mortality	3	2488	0.79	0.52‐1.20	47%	0.27
c) Ciprofloxacin vs no antibiotic						
Bacteremia	3	148	0.88	0.26‐2.97	24%	0.84
Neutropenic fever	3	203	0.85	0.58‐1.25	61%	0.41
Infection‐related mortality	4	253	0.65	0.18‐2.31	0%	0.51
Overall mortality	3	218	1.70	0.22‐13.01	43%	0.61
3. Fluoroquinolone vs non‐absorbable antibiotic						
Fever	3	235	0.98	0.91‐1.05	0%	0.50
Infection‐related mortality	3	360	0.43	0.18‐1.05	0%	0.06
B. Fluoroquinolone vs Trimethoprim‐sulfamethoxazole						
Bacteremia	7	583	0.86	0.48‐1.54	66%	0.60
Fever	3	291	0.65	0.31‐1.37	89%	0.26
Infection‐related mortality	6	541	1.10	0.50‐2.39	0%	0.82
Invasive fungal disease	6	541	0.78	0.35‐1.75	0%	0.55

Abbreviations: CI, confidence interval; RR, risk ratio.

a No antibiotic includes placebo and usual care (no antibiotic prophylaxis) control groups.

Table 3 shows the synthesized outcomes for non‐fluoroquinolone‐based comparisons. When compared to no antibiotic, trimethoprim‐sulfamethoxazole significantly reduced bacteremia (RR 0.59, 95% CI 0.41‐0.85) and infection‐related mortality (RR 0.61, 95% CI 0.39‐0.94) without significantly reducing overall mortality (RR 0.61, 95% CI 0.28‐1.33). However, trimethoprim‐sulfamethoxazole prophylaxis increased resistance to this agent in bacteremia isolates (RR 2.91, 95% CI 1.65‐5.12). Cephalosporin prophylaxis consisted of cefepime (n = 1) and ceftriaxone (n = 3); it significantly reduced bacteremia (RR 0.30, 95% CI 0.16‐0.58) and fever (RR 0.83, 95% CI 0.71‐0.98) but did not significantly reduce infection‐related mortality (RR 1.03, 95% CI 0.27‐3.95) or overall mortality (RR 1.58, 95% CI 0.72‐3.45). Glycopeptide prophylaxis did not significantly reduce bacteremia or infection‐related mortality. Finally, the addition of rifampin to fluoroquinolone significantly reduced the risk of bacteremia compared to fluoroquinolone alone (RR 0.36, 95% CI 0.17‐0.77).

**Table 3:** Synthesized Outcomes for Non‐Fluoroquinolone‐based Comparisons^a^

Comparison and outcomes	Number studies	Number patients	RR	95% CI	*I* ^2^	*P*
A. Trimethoprim‐sulfamethoxazole vs No Antibiotic						
Bacteremia	7	735	0.59	0.41‐0.85	0%	0.005
Fever	5	388	0.77	0.56‐1.07	91%	0.11
Infection‐related mortality	13	984	0.61	0.39‐0.94	0%	0.03
Overall mortality	5	268	0.61	0.28‐1.33	32%	0.21
Invasive fungal disease	7	744	1.19	0.43‐3.27	27%	0.74
Antibiotic resistance	5	68	2.91	1.65‐5.12	0%	0.0002
B. Cephalosporin vs No Antibiotic						
Bacteremia	4	337	0.30	0.16‐0.58	42%	0.0004
Fever	4	337	0.83	0.71‐0.98	65%	0.03
Infection‐related mortality	3	316	1.03	0.27‐3.95	0%	0.96
Overall mortality	3	272	1.58	0.72‐3.45	0%	0.26
C. Parenteral Glycopeptide vs No Antibiotic						
Bacteremia	3	170	0.45	0.08‐2.66	84%	0.38
Infection‐related mortality	3	273	1.13	0.30‐4.23	10%	0.85
D. Rifampin Plus Fluoroquinolone vs Fluoroquinolone^b^						
Bacteremia	3	236	0.36	0.17‐0.77	0%	0.008

Abbreviations: CI, confidence interval; RR, risk ratio.

a No antibiotic includes placebo and usual care (no antibiotic prophylaxis) control groups.

b No events for either infection‐related mortality or overall mortality for two of three studies.

Table 4 shows the stratified analyses for fluoroquinolone vs no antibiotic or nonabsorbable antibiotic for the two outcomes of bacteremia and overall mortality. Heterogeneity in the prophylaxis effect against bacteremia was not explained by treatment (chemotherapy, HSCT or both), age of participants (adult or pediatric), year of publication (early or late), risk of bacteremia in the control arm (low or high), adequate sequence generation or adequate allocation concealment. Similarly, heterogeneity in the treatment effect for overall mortality was not explained by treatment, year of publication, adequate sequence generation or adequate allocation concealment.

**Table 4:** Stratified analyses for fluoroquinolone vs no antibiotic or nonabsorbable antibiotic for bacteremia and overall mortality^a^

Subgroup	Number studies	Number patients	RR	95% CI	*I* ^2^	*P*
Outcome of bacteremia						
Treatment						Pint = 0.75
Chemotherapy only	8	606	0.46	0.30‐0.70	0%	0.0003
Stem cell transplantation only	3	187	0.48	0.13‐1.77	72%	0.27
Both chemotherapy and transplantation	3	1361	0.55	0.45‐0.67	0%	<0.0001
Age participants						Pint = 0.67
Adult	11	1396	0.54	0.36‐0.79	66%	0.002
Pediatric	2	708	0.66	0.27‐1.63	22%	0.37
Year of publication						Pint = 0.29
Earlier than 2000	8	554	0.46	0.31‐0.68	0%	0.0001
In or later than 2000	6	1600	0.63	0.42‐0.95	76%	0.03
Risk bacteremia in control group						Pint = 0.63
<27% (median value)	7	1065	0.52	0.39‐0.70	0%	<0.0001
≥27%	7	1089	0.60	0.38‐0.93	76%	0.02
Adequate sequence generation						Pint = 0.35
Yes	6	1570	0.62	0.40‐0.96	78%	0.03
No	8	584	0.47	0.32‐0.69	0%	0.0001
Adequate allocation concealment						Pint = 0.48
Yes	5	895	0.64	0.35‐1.17	76%	0.15
No	9	1259	0.51	0.41‐0.64	0%	<0.00001
Outcome of overall mortality						
Treatment						Pint = 0.27
Chemotherapy only	11	2551	0.95	0.70‐1.29	17%	0.74
Stem cell transplantation only	2	84	0.31	0.01‐7.45	NA^b^	0.47
Both chemotherapy and transplantation	4	1107	0.60	0.36‐1.00	0%	0.05
Year of Publication						Pint = 0.75
Earlier than 2000	9	724	0.89	0.57‐1.38	0%	0.59
In or later than 2000	8	3018	0.80	0.51‐1.26	44%	0.34
Adequate sequence generation						Pint = 0.14
Yes	6	2625	0.63	0.41‐0.97	0%	0.03
No	11	1117	0.97	0.67‐1.41	22%	0.87
Adequate allocation concealment						Pint = 0.89
Yes	6	1957	0.80	0.51‐1.26	0%	0.33
No	11	1785	0.83	0.56‐1.25	32%	0.37

Abbreviations: CI, confidence interval; Pint, P value for interaction, indicating subgroup heterogeneity; RR, risk ratio.

a No antibiotic includes placebo and usual care (no antibiotic prophylaxis) control groups.

b One study had zero events in both arms.

Potential publication bias was observed for the comparison of fluoroquinolone vs. no antibiotic or nonabsorbable antibiotic controls for the outcomes of bacteremia (Supplemental Appendix S3), fever (not shown) and overall mortality (Supplemental Appendix S4) but not infection‐related mortality (not shown). The comparison of trimethoprim‐sulfamethoxazole vs no antibiotic did not suggest publication bias for the outcome of infection‐related mortality, the only outcome amenable to funnel plot visualization. We applied the trim and fill approach for the comparison of fluoroquinolone vs no antibiotic or nonabsorbable antibiotic controls for the outcome of bacteremia. When the outlying study was removed and when a hypothetical negative study with equal weight was added, the resultant estimates remained significant (RR 0.57, 95% CI 0.42‐0.77 and RR 0.57, 95% CI 0.42‐0.78, respectively).

In terms of the network meta‐analysis, 33 studies reporting on bacteremia were included after limiting to studies that evaluated fluoroquinolone or cephalosporin in any arm and removing studies comparing the same specific antibiotic (for example, compared different doses of the same antibiotic). Supplemental Appendix S5 and Appendix S6 illustrate the networks and show direct and indirect comparisons available. In the comparison of fluoroquinolone vs. cephalosporin prophylaxis (Supplemental Appendix S5), there were no studies that directly compared these two antibiotic types. Cephalosporin, when compared to fluoroquinolone, did not significantly reduce bacteremia by network meta‐analysis (RR 0.58, 95% credible limit 0.27 to 1.2). In the comparison of levofloxacin vs ciprofloxacin (Supplemental Appendix S6), there were no studies that directly compared these two antibiotic types. Levofloxacin did not significantly reduce bacteremia when compared to ciprofloxacin by network meta‐analysis (RR 0.79, 95% credible limit 0.42 to 1.5).

## 4 DISCUSSION

In this systematic review, we found that fluoroquinolone prophylaxis was effective at reducing bacteremia, fever and infection‐related mortality without significantly increasing *C difficile* infection, invasive fungal disease or musculoskeletal adverse effects when compared to no antibiotic or nonabsorbable antibiotic controls. However, fluoroquinolone prophylaxis increased fluoroquinolone resistance in bacteremia isolates. We also found that at least once daily trimethoprim‐sulfamethoxazole prophylaxis was effective in reducing bacteremia and infection‐related mortality when compared to no antibiotic controls although it did increase trimethoprim‐sulfamethoxazole resistance in bacteremia isolates. Cephalosporin prophylaxis reduced bacteremia but did not significantly reduce infection‐related mortality, with antibiotic resistance not being evaluable. The fluoroquinolone prophylaxis effect was similar among sub‐groups evaluated related to bacteremia and overall survival. Finally, fluoroquinolone vs cephalosporin and levofloxacin vs ciprofloxacin had similar effects in terms of bacteremia prevention in network meta‐analysis.

Our systematic review is important as it not only includes the most recent randomized trials, but in addition, evaluates heterogeneity in the fluoroquinolone prophylaxis effect and includes a network meta‐analysis to contrast therapies in which no direct comparative trials exist. Prior systematic reviews focused on specific patient populations such as those with hematological malignancy^14^ and HSCT^15^ or specific antibiotic classes such as fluoroquinolone.^16^ In contrast, our review included all systemic antibiotics and all cancer therapies including HSCT. The only published broadly inclusive systematic review does not include data from trials published in the last eight years (included studies until 2010).^7^

We did not find that the following explained heterogeneity in the effect of fluoroquinolone prophylaxis to reduce bacteremia: patient group (chemotherapy, HSCT or both), age (adult or pediatric), year of publication (early or late), or risk of bacteremia in the control arm (low or high). This suggests that prophylaxis can be considered in a broad group of patients. However, it is important to note that we evaluated RRs in this study and that as the prevalence of bacteremia decreases, the RR associated with prophylaxis may remain constant but the absolute risk reduction could be diminished to the point that prophylaxis is no longer worthwhile. For example, a RR of 0.5 represents both decreasing bacteremia risk from 80% to 40% (probably worthwhile) and decreasing risk from 0.2% to 0.1% (probably not worthwhile).

We found that fluoroquinolone prophylaxis did not reduce overall mortality either among the entire cohort or among different sub‐groups. We also found evidence of publication bias in this outcome, further supporting a lack of impact on survival. In contrast to our results, one previous meta‐analysis by Gafter‐Gvili demonstrated that fluoroquinolone prophylaxis significantly reduced overall mortality.^7^ Interestingly, three other systematic reviews have not shown a statistically significant reduction in overall mortality associated with fluoroquinolone prophylaxis.^14^, ^15^, ^16^ Differences between the Gafter‐Gvili review and ours include the following items. Our review included more recent studies and restricted the fluoroquinolone analysis to studies that only administered fluoroquinolone in the intervention group. Conversely, the Gafter‐Gvili review included combination antibiotics with a fluoroquinolone as one component of the intervention. This different definition of the intervention group is important as our systematic review showed that the combination of rifampin and fluoroquinolone was better than fluoroquinolone alone in reducing bacteremia.

In our conventional meta‐analysis, the effect of levofloxacin vs no antibiotic to reduce bacteremia was RR 0.54 while the effect of ciprofloxacin vs no antibiotic to reduce bacteremia was RR 0.88. The P value for interaction for the analysis of fluoroquinolone type was not significant. In support of this finding, network meta‐analysis failed to show a difference between levofloxacin and ciprofloxacin. However, it is important to emphasize that network meta‐analysis may be problematic when patients, controls or interventions are heterogeneous.^17^ While clear differences in the population between the levofloxacin and ciprofloxacin studies were not evident, we cannot exclude incoherence, or important differences between direct and indirect estimates, as we lacked direct comparative data.

It is important to note that these studies evaluated the efficacy and adverse effects of systemic antibiotic prophylaxis when administered within a finite time frame of a clinical trial among enrolled participants. An important knowledge gap is the long‐term impact on effectiveness, adverse effects, and resistance outcomes when prophylaxis is administered as routine care over multiple treatment cycles in a universal prophylaxis strategy. These outcomes will be important to measure in future research.

The strengths of our review include its timely and comprehensive nature as well as its rigorous methodology. Furthermore, inclusion of a network meta‐analysis is another strength. However, this review must be interpreted in light of its weaknesses. First, we did not have access to individual level data, which could have allowed better identification of sub‐groups more likely to benefit (or more likely to be harmed) from prophylaxis. Second, as with all meta‐analysis, there is the potential for bias in terms of which outcomes were reported in individual trials. Third, some syntheses such as the evaluation of *C difficile* infection included few or no studies, thus limiting or precluding the ability to detect an effect. Fourth, we were not able to synthesize results by neoplasm type based upon how studies were conducted and reported. Finally, we measured antibiotic resistance only against the intervention being evaluated. There are two important issues with this approach. First, we did not measure if resistance rates in the control group were increased, which is plausible with greater environmental exposure to an antibiotic. Second, we did not evaluate resistance to other antibiotics in either group.

In conclusion, fluoroquinolone, trimethoprim‐sulfamethoxazole and cephalosporin prophylaxis reduced bacteremia but did not significantly reduce overall mortality. A clinical practice guideline to facilitate prophylactic antibiotic decision‐making is required.

## 5 AUTHOR CONTIBUTIONS

GE, PDR and LS conceptualized the study, designed the study, collected the data, analyzed the data, and wrote the manuscript; JPM and GT conceptualized and conducted the network meta‐analysis and analyzed this data. All authors critically revised the manuscript for important content. All authors approve the final version of the manuscript.

## CONFLICTS OF INTERESTS

GE, PDR, JPDM, SA, RAA, LLD, BP, SC, GT and LS declare no potential conflicts of interest. BTF's institution receives research funding from Pfizer and Merck, and he serves on a Data Safety Monitoring Board for Astellas. TL has received research grants from Gilead Sciences, is a consultant to Astellas, Basilea, Gilead Sciences, and Merck/MSD, and served at the speaker's bureau of Astellas, Gilead Sciences, Merck/MSD, and Pfizer.

## Data Availability

The study was based entirely on previously published data.
