# Supplementary material for: Efficacy of antibiotic prophylaxis in patients with cancer and hematopoietic stem cell transplantation recipients: A systematic review of randomized trials
Source: Cancer Med. 2019 Jul 5;8(10):4536–46. doi: 10.1002/cam4.2395 (PMC6712447; doi:10.1002/cam4.2395)
Supplement: Supplementary file 1 [file CAM4-8-4536-s001.docx]

**Appendix 1: Search Strategies**

OvidSP **MEDLINE** to November Week 2 2018

| **Set** | **History** |
| --- | --- |
| 1 | exp neoplasms/ |
| 2 | (cancer* or oncol* or tumour* or tumor* or malignan* or neoplas* or sarcom* or blastoma* or neuroblastoma* or leukem* or leukaem* or carcinoma* or lymphoma* or adenocarcinoma* or hodgkin*).ti,ab,kf. |
| 3 | exp Antineoplastic Agents/ |
| 4 | antineoplastic protocols/ or antineoplastic combined chemotherapy protocols/ |
| 5 | ((adjuvant adj2 chemotherap*) or chemoradiotherap* or radiochemotherap* or chemotherap* or radiation*).ti,ab,kf. |
| 6 | radiation dosage/ or dose-response relationship, radiation/ or Radiometry/ or Radiotherapy Dosage/ or (((gray or sievert) adj2 unit*) or (radiation adj2 (dosage* or dose or dosing)) or "gy radiation" or "radiation dose-response").mp. or chemoradiotherapy/ or chemoradiotherapy, adjuvant/ or Radiotherapy, Adjuvant/ or rt.fs. or radiotherapy/ |
| 7 | transplantation, autologous/ or transplantation, heterologous/ or transplantation, heterotopic/ or exp transplantation, homologous/ or stem cell transplantation/ or allogeneic stem cell transplantation/ or allogeneic hematopoietic stem cell transplantation/ or allogeneic peripheral blood stem cell transplantation/ |
| 8 | bone marrow transplantation/ |
| 9 | (stem adj3 cell adj3 (transplant* or graft*)).ti,ab,kf. |
| 10 | (hsct or bmt or (bone adj2 marrow adj (transplant* or graft*))).ti,ab,kf. |
| 11 | ((autologous or hematopoietic or heterologous) adj3 (transplant* or graft*)).ti,ab,kf. |
| 12 | agranulocytosis/ or neutropenia/ or febrile neutropenia/ or chemotherapy-induced febrile neutropenia/ |
| 13 | (neutropen* or neutropaen* or agranulocyto* or granulocytopenic*).ti,ab,kf. |
| 14 | Immunocompromised Host/ |
| 15 | (immunocompromis* or immunocompromiz*).ti,ab,kf. |
| 16 | or/1-15 |
| 17 | antibiotic prophylaxis/ |
| 18 | chemoprevention/ or (prophylax* or prophylactic* or chemoprevent* or chemoprophylactic* or chemoprophylax* or prevent* or "pre-emptive*").ti,ab,kf. |
| 19 | exp antibacterial agents/ or (antibiotic* or antibacterial* or "anti-microbial*" or "anti-bacterial*").ti,ab,kf. |
| 20 | anti-infective agents/ |
| 21 | sulfamethoxazole/ or trimethoprim, sulfamethoxazole drug combination/ or Trimethoprim/ |
| 22 | fluoroquinolones/ or ciprofloxacin/ or fleroxacin/ or enoxacin/ or norfloxacin/ or ofloxacin/ or levofloxacin/ or pefloxacin/ |
| 23 | exp Cephalosporins/ |
| 24 | Rifampin/ |
| 25 | polymyxins/ or colistin/ or polymyxin b/ |
| 26 | exp Aminoglycosides/ |
| 27 | exp Quinolones/ |
| 28 | exp beta-lactams/ or exp cephalosporins/ or clavulanic acids/ or clavulanic acid/ or amoxicillin-potassium clavulanate combination/ |
| 29 | Cefepime.mp. |
| 30 | exp Erythromycin/ |
| 31 | (Phenethicillin or Roxythromycin).mp. |
| 32 | Tosufloxacin.mp. |
| 33 | Cephalotin.mp. |
| 34 | exp Neomycin/ |
| 35 | or/19-35 |
| 36 | 18 and 35 |
| 37 | 17 or 36 |
| 38 | 16 and 37 |
| 39 | ("clinical trial, all" or clinical trial).pt. or clinical trials as topic/ |
| 40 | clinical trial, phase i.pt. or clinical trials, phase i as topic/ |
| 41 | clinical trial, phase ii.pt. or clinical trials, phase ii as topic/ |
| 42 | clinical trial, phase iii.pt. or clinical trials, phase iii as topic/ |
| 43 | clinical trial, phase iv.pt. or clinical trials, phase iv as topic/ |
| 44 | controlled clinical trial.pt. or controlled clinical trials as topic/ |
| 45 | meta-analysis.pt. or meta-analysis as topic/ |
| 46 | multicenter study.pt. or multicenter studies as topic/ |
| 47 | randomized controlled trial.pt. or randomized controlled trials as topic/ |
| 48 | pragmatic clinical trial.pt. or Pragmatic Clinical Trials as Topic/ or ((preference or practical or pragmatic or "real world" or naturalistic) adj5 trial*).ti,ab. |
| 49 | Comparative Effectiveness Research/ or ((comparative adj2 effectiveness) or (CER adj5 (research* or method* or framework* or compari* or statement*))).ti,ab. |
| 50 | (randomized or placebo or randomly or trial or groups).ab. |
| 51 | or/39-50 |
| 52 | 38 and 51 |
| 53 | limit 52 to yr="1980 -Current" |
| 54 | limit 53 to humans |
| 55 | limit 53 to animals |
| 56 | 53 not (54 or 55) |
| 57 | 54 or 56 |

**MEDLINE in Process**

OvidSP **MEDLINE**(R) In-Process & Other Non-Indexed Citations November 15, 2018

| **Set** | **History** |
| --- | --- |
| 1 | (cancer* or oncol* or tumour* or tumor* or malignan* or neoplas* or sarcom* or blastoma* or neuroblastoma* or leukem* or leukaem* or carcinoma* or lymphoma* or adenocarcinoma* or hodgkin*).ti,ab,kf. |
| 2 | (Antineoplastic* or "anti-neoplastic*").mp. |
| 3 | ((adjuvant adj2 chemotherap*) or chemoradiotherap* or radiochemotherap* or chemotherap* or radiation*).ti,ab,kf. |
| 4 | (((gray or sievert) adj2 unit*) or (radiation adj2 (dosage* or dose or dosing)) or ("gy radiation" or "radiation dose-response")).ti,ab,kf. |
| 5 | (stem adj3 cell adj3 (transplant* or graft*)).ti,ab,kf. |
| 6 | ((autologous or hematopoietic or heterologous) adj3 (transplant* or graft*)).ti,ab,kf. |
| 7 | (neutropen* or neutropaen* or agranulocyto* or granulocytopenic*).ti,ab,kf. |
| 8 | (immunocompromis* or immunocompromiz*).ti,ab,kf. |
| 9 | (hsct or bmt or (bone adj2 marrow adj (transplant* or graft*))).ti,ab,kf. |
| 10 | or/1-9 |
| 11 | (chemoprevent* or prophylax* or prophylactic* or chemoprevent* or chemoprophylactic* or chemoprophylax* or prevent* or "pre-emptive*").ti,ab,kf. |
| 12 | (antibiotic* or antiinfective or "anti-infective" or antibacterial* or "anti-microbial*" or "anti-bacterial*").ti,ab,kf. |
| 13 | (sulfamethoxazole or trimethoprim or Amoxicillin or clavulanate or Cefepime or Ceftriaxone or Ciprofloxacin or Enoxacin or Erythromycin or Imipenem or Levofloxacin or Nalidixic or Norfloxacin or Norfloxacin or Ofloxacin or Pefloxacin or Penicillin* or Phenethicillin or Rifampin or Roxythromycin or Teicoplanin or Ticarcillin* or Tobramycin or tosufloxacin or Vancomycin).mp. |
| 14 | (fluoroquinolone* or ciprofloxacin or fleroxacin or enoxacin or norfloxacin or ofloxacin or levofloxacin or pefloxacin).mp. |
| 15 | (Rifampin or Cephalosporin* or polymyxin* or colistin).mp. |
| 16 | (Aminoglycoside* or Quinolone* or "beta-lactams" or "beta lactams" or Cefepime or Erythromycin*).mp. |
| 17 | (Phenethicillin or Roxythromycin).mp. |
| 18 | (Tosufloxacin or Cephalotin).mp. |
| 19 | Neomycin*.mp. |
| 20 | or/12-19 |
| 21 | 10 and 20 |
| 22 | (randomized or placebo or randomly or trial or groups).ab. |
| 23 | ("meta-analys*" or metaanalys* or multicent* or rct or rcts).ti,ab,kf. |
| 24 | or/22-23 |
| 25 | 21 and 24 |

**MEDLINE Epub Ahead of Print**

OvidSP **MEDLINE(R)** Epub Ahead of Print November 15, 2018

| **Set** | **History** |
| --- | --- |
| 1 | (cancer* or oncol* or tumour* or tumor* or malignan* or neoplas* or sarcom* or blastoma* or neuroblastoma* or leukem* or leukaem* or carcinoma* or lymphoma* or adenocarcinoma* or hodgkin*).ti,ab,kf. |
| 2 | (Antineoplastic* or "anti-neoplastic*").mp. |
| 3 | ((adjuvant adj2 chemotherap*) or chemoradiotherap* or radiochemotherap* or chemotherap* or radiation*).ti,ab,kf. |
| 4 | (((gray or sievert) adj2 unit*) or (radiation adj2 (dosage* or dose or dosing)) or ("gy radiation" or "radiation dose-response")).ti,ab,kf. |
| 5 | (stem adj3 cell adj3 (transplant* or graft*)).ti,ab,kf. |
| 6 | ((autologous or hematopoietic or heterologous) adj3 (transplant* or graft*)).ti,ab,kf. |
| 7 | (neutropen* or neutropaen* or agranulocyto* or granulocytopenic*).ti,ab,kf. |
| 8 | (immunocompromis* or immunocompromiz*).ti,ab,kf. |
| 9 | (hsct or bmt or (bone adj2 marrow adj (transplant* or graft*))).ti,ab,kf. |
| 10 | or/1-9 |
| 11 | (chemoprevent* or prophylax* or prophylactic* or chemoprevent* or chemoprophylactic* or chemoprophylax* or prevent* or "pre-emptive*").ti,ab,kf. |
| 12 | (antibiotic* or antiinfective or "anti-infective" or antibacterial* or "anti-microbial*" or "anti-bacterial*").ti,ab,kf. |
| 13 | (sulfamethoxazole or trimethoprim or Amoxicillin or clavulanate or Cefepime or Ceftriaxone or Ciprofloxacin or Enoxacin or Erythromycin or Imipenem or Levofloxacin or Nalidixic or Norfloxacin or Norfloxacin or Ofloxacin or Pefloxacin or Penicillin* or Phenethicillin or Rifampin or Roxythromycin or Teicoplanin or Ticarcillin* or Tobramycin or tosufloxacin or Vancomycin).mp. |
| 14 | (fluoroquinolone* or ciprofloxacin or fleroxacin or enoxacin or norfloxacin or ofloxacin or levofloxacin or pefloxacin).mp. |
| 15 | (Rifampin or Cephalosporin* or polymyxin* or colistin).mp. |
| 16 | (Aminoglycoside* or Quinolone* or "beta-lactams" or "beta lactams" or Cefepime or Erythromycin*).mp. |
| 17 | (Phenethicillin or Roxythromycin).mp. |
| 18 | (Tosufloxacin or Cephalotin).mp. |
| 19 | Neomycin*.mp. |
| 20 | or/12-19 |
| 21 | 10 and 20 |
| 22 | (randomized or placebo or randomly or trial or groups).ab. |
| 23 | ("meta-analys*" or metaanalys* or multicent* or rct or rcts).ti,ab,kf. |
| 24 | or/22-23 |
| 25 | 21 and 24 |

**EMBASE**

OvidSP **Embase** 1980 to 2018 Week 46

| **Set** | **History** |
| --- | --- |
| 1 | exp neoplasm/ |
| 2 | (cancer* or oncol* or tumour* or tumor* or malignan* or neoplas* or sarcom* or blastoma* or neuroblastoma* or leukem* or leukaem* or carcinoma* or lymphoma* or adenocarcinoma* or hodgkin*).ti,ab,kw. |
| 3 | exp Antineoplastic Agent/ |
| 4 | (antineoplas* adj5 protocol*).ti,ab,kw. |
| 5 | ((adjuvant adj2 chemotherap*) or chemoradiotherap* or radiochemotherap* or chemotherap* or radiation*).ti,ab,kw. |
| 6 | radiotherapy/ or blood radiation/ or chemoradiotherapy/ or adjuvant chemoradiotherapy/ or radiotherapy/ or blood radiation/ or exp chemoradiotherapy/ or exp cobalt therapy/ or image guided radiotherapy/ or intensity modulated radiation therapy/ or intraoperative radiotherapy/ or megavoltage radiotherapy/ or radiation depth dose/ or radiation dose/ or radiation dose escalation/ or radiation dose fractionation/ or radiation dose reduction/ or radiation response/ or radioimmunotherapy/ or radiation measurement/ or dosimetry/ or radiometry/ |
| 7 | (((gray or sievert) adj2 unit*) or (radiation adj2 (dosage* or dose or dosing)) or "gy radiation" or "radiation dose-response").mp. or rt.fs. or ((adjuvant adj2 chemotherap*) or chemoradiotherap* or radiochemotherap* or chemotherap* or radiation*).ti,ab,kw. |
| 8 | stem cell transplantation/ or exp allogeneic stem cell transplantation/ or autologous stem cell transplantation/ or exp hematopoietic stem cell transplantation/ or mesenchymal stem cell transplantation/ or bone marrow transplantation/ or tissue transplantation/ or allogenic bone marrow transplantation/ or autologous bone marrow transplantation/ or bone marrow purging/ or bone marrow rescue/ |
| 9 | transplantation/ or allotransplantation/ or autotransplantation/ or heterotopic transplantation/ |
| 10 | (stem adj3 cell adj3 (transplant* or graft*)).ti,ab,kw. |
| 11 | (hsct or bmt or (bone adj2 marrow adj (transplant* or graft*))).ti,ab,kw. |
| 12 | ((autologous or hematopoietic or heterologous) adj3 (transplant* or graft*)).ti,ab,kw. |
| 13 | agranulocytosis/ or neutropenia/ or febrile neutropenia/ or leukopenia/ |
| 14 | (neutropen* or neutropaen* or agranulocyto* or granulocytopenic*).ti,ab,kw. |
| 15 | immunosuppressive treatment/ or immunocompromised patient/ |
| 16 | (immunocompromis* or immunocompromiz*).ti,ab,kw. |
| 17 | or/1-16 |
| 18 | antibiotic prophylaxis/ |
| 19 | prophylaxis/ or chemoprophylaxis/ or (prophylax* or prophylactic* or chemoprevent* or chemoprophylactic* or chemoprophylax* or prevent* or "pre-emptive*").ti,ab,kw. |
| 20 | exp antibiotic agent/ |
| 21 | (antibiotic* or antibacterial* or "anti-microbial*" or "anti-bacterial*").ti,ab,kw. |
| 22 | antiinfective agent/ |
| 23 | sulfamethoxazole/ |
| 24 | trimethoprim derivative/ |
| 25 | quinolone derivative/ or "1 (3,4 dichlorobenzyl) 1,2 dihydro 3 methyl 2 oxo 4 quinolineacetic acid"/ or "1 [1 [4 (3 acetamidopropoxy)benzoyl] 4 piperidyl] 1,2,3,4 tetrahydro 2 quinolinone"/ or "1 [3 [4 (3 chlorophenyl) 1 piperazinyl]propyl] 3,4 dihydro 5 methoxy 2(1h) quinolinone"/ or "1 cyclopropyl 6 fluoro 1,4 dihydro 4 oxo 7 (1 pyrrolidinyl) 3 quinolinecarboxylic acid"/ or "1 cyclopropyl 6 fluoro 1,4 dihydro 8 methoxy 4 oxo 7 (1 piperazinyl) 3 quinolinecarboxylic acid"/ or 1 cyclopropyl 6 fluoro 1,4 dihydro 8 methyl 7 morpholino 4 oxo 3 quinolinecarboxylic acid/ or "1 cyclopropyl 7 (2,6 dimethyl 4 pyridyl) 6 fluoro 1,4 dihydro 4 oxo 3 quinolinecarboxylic acid"/ or "1 cyclopropyl 7 (3 ethyl 1 piperazinyl) 6 fluoro 1,4 dihydro 4 oxo 3 quinolinecarboxylic acid"/ or "1 cyclopropyl 7 (3 ethyl 1 piperazinyl) 6 fluoro 1,4 dihydro 8 methoxy 4 oxo 3 quinolinecarboxylic acid"/ or "1 cyclopropyl 7 [3 [(ethylamino)methyl] 1 pyrrolidinyl] 6,8 difluoro 1,4 dihydro 4 oxo 3 quinolinecarboxylic acid"/ or 1 ethyl 1,4 dihydro 4 oxo 3 quinolinecarboxylic acid derivative/ or "1 ethyl 6 [[3 fluoro 5 (3,4,5,6 tetrahydro 4 methoxy 2h pyran 4 yl)]phenoxymethyl] 2 quinolone"/ or "1 ethyl 6 fluoro 1,4 dihydro 7 (2 methyl 5 oxazolyl) 4 oxo 3 quinolinecarboxylic acid"/ or 1 ethyl 6 fluoro 1,4 dihydro 7 methyl 4 oxo 3 quinolinecarboxylic acid/ or "1 ethyl 6,8 difluoro 1,4 dihydro 4 oxo 7 (4 pyridyl) 3 quinolinecarboxylic acid"/ or "1 ethyl 6,8 difluoro 1,4 dihydro 7 (2 hydroxyethylamino) 4 oxo 3 quinolinecarboxylic acid"/ or "3,4 dihydro 6 [3 [1 (2 methylphenyl) 2 imidazolylsulfinyl]propoxy] 2(1h) quinolinone"/ or "3,4 dihydro 6 [4 (4 oxo 4 phenylbutyl) 1 piperazinylcarbonyl]carbostyril"/ or 4 quinolone derivative/ or "5 amino 7 (3 amino 1 pyrrolidinyl) 1 cyclopropyl 6,8 difluoro 1,4 dihydro 4 oxo 3 quinolinecarboxylic acid"/ or "6 (4 acetyl 2 methyl 1 imidazolyl) 8 methylcarbostyril"/ or "6 [[3 fluoro 5 (3,4,5,6 tetrahydro 4 methoxy 2h pyran 4 yl)]phenoxymethyl] 1 methyl 2 quinolone"/ or "6 fluoro 1 (2 fluoroethyl) 1,4 dihydro 7 (4 methyl 1 piperazinyl) 4 oxo 3 quinolinecarboxylic acid"/ or "6 fluoro 1,4 dihydro 1 methylamino 7 (1 piperazinyl) 4 oxo 3 quinolinecarboxylic acid"/ or 6 fluoro 1,4 dihydro 4 oxo 3 quinolinecarboxylic acid derivative/ or "7 (2 aminoethylamino) 1 cyclopropyl 6 fluoro 1,4 dihydro 4 oxo 3 quinolinecarboxylic acid"/ or "7 (2 aminomethyl 4 morpholinyl) 1 cyclopropyl 6,8 difluoro 1,4 dihydro 4 oxo 3 quinolinecarboxylic acid"/ or "7 (3 amino 1 pyrrolidinyl) 1 cyclopropyl 6,8 difluoro 1,4 dihydro 4 oxo 3 quinolinecarboxylic acid"/ or "7 (3 amino 3 methyl 1 azetidinyl) 1 cyclopropyl 6,8 difluoro 1,4 dihydro 4 oxo 3 quinolinecarboxylic acid"/ or "7 (3 amino 4 methyl 1 pyrrolidinyl) 1 (2,4 difluorophenyl) 6 fluoro 1,4 dihydro 4 oxo 3 quinolinecarboxylic acid"/ or "7 (3 amino 4 methyl 1 pyrrolidinyl) 1 cyclopropyl 6 fluoro 1,4 dihydro 8 methyl 4 oxo 3 quinolinecarboxylic acid"/ or "7 (3 aminomethyl 3 fluoromethyl 1 pyrrolidinyl) 1 cyclopropyl 6 fluoro 1,4 dihydro 8 methoxy 4 oxo 3 quinolinecarboxylic acid"/ or "7 (7 amino 7 methyl 5 azaspiro[2.4]heptan 5 yl) 6 fluoro 1 (2 fluorocyclopropyl) 1,4 dihydro 8 methoxy 4 oxo 3 quinolinecarboxylic acid"/ or "7 [3 (1 aminocyclopropyl) 1 pyrrolidinyl] 1 (2 fluorocyclopropyl) 1,4 dihydro 8 methoxy 4 oxo 3 quinolinecarboxylic acid"/ or "7 [3 [4 (2,3 dimethylphenyl) 1 piperazinyl]propoxy] 2(1h) quinolinone"/ or "7 chloro 4 hydroxy 3 (3 phenoxyphenyl) 2 quinolone"/ or "7 chloro 4 hydroxy 3 [3 (4 methoxybenzyl)phenyl] 2 quinolone"/ or "8 chloro 1 cyclopropyl 7 (2,8 diazabicyclo[4.3.0]non 8 yl) 6 fluoro 1,4 dihydro 4 oxo 3 quinolinecarboxylic acid"/ or "8 difluoromethoxy 1 ethyl 6 fluoro 1,4 dihydro 7 [4 (2 methoxyphenyl) 1 piperazinyl] 4 oxo 3 quinolinecarboxylic acid"/ or 8 hydroxycarteolol/ or "8 methyl 3 (4 methyl 1 piperazinyl)carbostyril"/ or acorafloxacin/ or amifloxacin piperazinyl n oxide/ or aripiprazole/ or aripiprazole lauroxil/ or balofloxacin/ or besifloxacin/ or binfloxacin/ or brefonalol/ or brexpiprazole/ or cadrofloxacin/ or carbostyril derivative/ or carmoterol/ or carteolol/ or cetefloxacin/ or ciprofloxacin/ or ciprofloxacin plus hydrocortisone/ or clinafloxacin/ or danofloxacin/ or delafloxacin/ or difloxacin/ or edulinine/ or enrofloxacin/ or fandofloxacin/ or finafloxacin/ or fleroxacin/ or flosequinan/ or flosequinan sulfone/ or garenoxacin/ or gatifloxacin/ or grepafloxacin/ or indacaterol/ or indacaterol plus mometasone furoate/ or irloxacin/ or ivacaftor/ or ivacaftor plus lumacaftor/ or ivacaftor plus tezacaftor/ or lascufloxacin/ or lavamilast/ or lomefloxacin/ or merafloxacin/ or miloxacin/ or mometasone furoate plus orbifloxacin plus posaconazole/ or moxifloxacin/ or nanterinone/ or nemonoxacin/ or nequinate/ or nordifloxacin/ or norfleroxacin/ or norfloxacin/ or olamufloxacin/ or orbifloxacin/ or oxociprofloxacin/ or oxolinic acid/ or ozenoxacin/ or pefloxacin/ or piperanometozine/ or pradofloxacin/ or premafloxacin/ or quinolone/ or rebamipide/ or rosoxacin/ or rufloxacin/ or sitafloxacin/ or sparfloxacin/ or temafloxacin/ or toborinone/ |
| 26 | exp quinolone derivative/ |
| 27 | exp quinoline derived antiinfective agent/ |
| 28 | exp cephalosporin derivative/ |
| 29 | rifampicin/ |
| 30 | polymyxin/ or polymyxin b/ or colistin/ |
| 31 | exp aminoglycoside antibiotic agent/ |
| 32 | exp beta lactam antibiotic/ |
| 33 | amoxicillin plus clavulanic acid/ |
| 34 | erythromycin/ |
| 35 | imipenem/ |
| 36 | exp penicillin derivative/ |
| 37 | isoniazid/ |
| 38 | Roxythromycin.mp. |
| 39 | teicoplanin/ |
| 40 | amphotericin/ or amphotericin a/ or amphotericin b/ |
| 41 | or/20-40 |
| 42 | 19 and 41 |
| 43 | 18 or 42 |
| 44 | 17 and 43 |
| 45 | limit 44 to (randomized controlled trial or controlled clinical trial) |
| 46 | randomized controlled trial/ |
| 47 | double-blind procedure/ |
| 48 | single-blind procedure/ |
| 49 | crossover-procedure/ |
| 50 | random*.ti,ab,kw. |
| 51 | placebo.ti,ab,kw. |
| 52 | factorial*.ti,ab,kw. |
| 53 | crossover*.ti,ab,kw. |
| 54 | "cross over".ti,ab,kw. |
| 55 | "cross-over*".ti,ab,kw. |
| 56 | trial.ab. |
| 57 | trial.ab. |
| 58 | groups.ab. |
| 59 | (doubl* adj5 blind*).ti,ab,kw. |
| 60 | (singl* adj5 blind*).ti,ab,kw. |
| 61 | assign*.ti,ab,kw. |
| 62 | allocat*.ti,ab,kw. |
| 63 | volunteer*.ti,ab,kw. |
| 64 | or/46-63 |
| 65 | 45 or (44 and 64) |
| 66 | limit 65 to human |
| 67 | limit 65 to animals |
| 68 | limit 65 to animal studies |
| 69 | 67 or 68 |
| 70 | 65 not (66 or 69) |
| 71 | 65 or 70 |
| 72 | limit 71 to (abstract report or conference abstract) |
| 73 | 71 not 72 |

**Cochrane**

**Wiley Cochrane** Issue 11 of 12, November 2018

| **Set** | **History** |
| --- | --- |
| 1 | MeSH descriptor: [Neoplasms] explode all trees |
| 2 | cancer* or oncol* or tumour* or tumor* or malignan* or neoplas* or sarcom* or blastoma* or neuroblastoma* or leukem* or leukaem* or carcinoma* or lymphoma* or adenocarcinoma* or hodgkin* |
| 3 | MeSH descriptor: [Antineoplastic Agents] explode all trees |
| 4 | MeSH descriptor: [Antineoplastic Protocols] explode all trees |
| 5 | (adjuvant near/2 chemotherap*) or chemoradiotherap* or radiochemotherap* or chemotherap* or radiation* |
| 6 | MeSH descriptor: [Radiometry] explode all trees |
| 7 | ((gray or sievert) near/2 unit*) or (radiation near/2 (dosage* or dose or dosing)) or "gy radiation" or "radiation dose-response" |
| 8 | MeSH descriptor: [Chemoradiotherapy] explode all trees |
| 9 | MeSH descriptor: [Radiotherapy, Adjuvant] this term only |
| 10 | Any MeSH descriptor with qualifier(s): [Radiotherapy - RT] |
| 11 | MeSH descriptor: [Radiotherapy] this term only |
| 12 | MeSH descriptor: [Transplantation, Autologous] this term only |
| 13 | MeSH descriptor: [Transplantation, Heterologous] explode all trees |
| 14 | MeSH descriptor: [Transplantation, Homologous] explode all trees |
| 15 | MeSH descriptor: [Transplantation, Heterotopic] explode all trees |
| 16 | MeSH descriptor: [Bone Marrow Transplantation] this term only |
| 17 | MeSH descriptor: [Stem Cell Transplantation] explode all trees |
| 18 | (stem near/3 cell near/3 (transplant* or graft*)) |
| 19 | hsct or bmt or (bone near/2 marrow near/2 (transplant* or graft*)) |
| 20 | (autologous or hematopoietic or heterologous) near/3 (transplant* or graft*) |
| 21 | MeSH descriptor: [Agranulocytosis] explode all trees |
| 22 | neutropen* or neutropaen* or agranulocyto* or granulocytopenic* |
| 23 | MeSH descriptor: [Immunocompromised Host] this term only |
| 24 | immunocompromis* or immunocompromiz* |
| 25 | {or #1-#24} |
| 26 | MeSH descriptor: [Antibiotic Prophylaxis] this term only |
| 27 | MeSH descriptor: [Chemoprevention] this term only |
| 28 | prophylax* or prophylactic* or chemoprevent* or chemoprophylactic* or chemoprophylax* or prevent* or "pre-emptive*" |
| 29 | #27 or #28 |
| 30 | MeSH descriptor: [Anti-Bacterial Agents] explode all trees |
| 31 | MeSH descriptor: [Anti-Infective Agents] explode all trees |
| 32 | antibiotic* or antibacterial* or "anti-microbial*" or "anti-bacterial*" |
| 33 | MeSH descriptor: [Sulfamethoxazole] explode all trees |
| 34 | MeSH descriptor: [Trimethoprim] this term only |
| 35 | MeSH descriptor: [Fluoroquinolones] explode all trees |
| 36 | MeSH descriptor: [Cephalosporins] explode all trees |
| 37 | MeSH descriptor: [Rifampin] this term only |
| 38 | MeSH descriptor: [Polymyxins] explode all trees |
| 39 | MeSH descriptor: [Aminoglycosides] explode all trees |
| 40 | MeSH descriptor: [Quinolones] explode all trees |
| 41 | MeSH descriptor: [beta-Lactams] explode all trees |
| 42 | Cefepime |
| 43 | MeSH descriptor: [Erythromycin] explode all trees |
| 44 | Phenethicillin or Roxythro or Tosufloxacin or Cephalotin |
| 45 | MeSH descriptor: [Neomycin] explode all trees |
| 46 | {or #30-#45} |
| 47 | #26 or (#29 and #46) |
| 48 | #25 and #47 Publication Year from 1980 to 2018 |

**Supplemental Appendix 2: Details of Included Studies (N=113)**

| **Author** | **Year Pub** | **Intervention Group** | **Control Group*** | **No. Trial Arms** | **Country** | **Age Group** | **Age Range in Years** | **Treatment Group** | **Cancer Diagnosis or HSCT Type** | **No. Randomized** |
| --- | --- | --- | --- | --- | --- | --- | --- | --- | --- | --- |
| **Fluoroquinolone vs. No Antibiotic or Non-absorbable Antibiotics** | | | | | | | | | | |
| Alexander^1^ | 2018 | Levofloxacin | No antibiotic | 2 | US/Canada | Child | 3-16 | Both | >1 type | 624 |
| Laoprasopwattana^2^ | 2013 | Ciprofloxacin | Placebo | 2 | Thailand | Child | 0.25-18 | Cancer | >1 type | 95 |
| Widjjanto^3^ | 2013 | Ciprofloxacin | Placebo | 2 | Indonesia | Child | 1-14 | Cancer | Hematologic malignancy | 110 |
| Vehreschild^4^ | 2012 | Moxifloxacin | Placebo | 2 | Germany | Adult | 21-70 | HSCT | Autologous transplant | 68 |
| Vesole^5^ | 2012 | Ciprofloxacin or Ofloxacin | No antibiotic | 3 | US | Adult | NR | Cancer | Hematologic malignancy | 212 |
| Schuette^6^ | 2011 | Levofloxacin | Placebo | 2 | Germany | Adult | 59-83 | Cancer | Solid tumor | 192 |
| Rahman^7^ | 2009 | Levofloxacin | Placebo | 2 | Bangladesh | Adult | NR | Cancer | Hematologic malignancy | 80 |
| Bucaneve^8^ | 2005 | Levofloxacin | Placebo | 2 | Italy | Adult | 18-75 | Both | >1 type | 760 |
| Cullen^9^ | 2005 | Levofloxacin | Placebo | 2 | UK | Adult | 16-60 | Cancer | >1 type | 1565 |
| Nenova^10^ | 2001 | Ciprofloxacin or Enoxacin or Norfloxacin or Pefloxacin | Placebo | 2 | Bulgaria | Adult | 16-80 | Cancer | Hematologic malignancy | 71 |
| Prentice^11^ | 2001 | Ciprofloxacin | Non-absorbable | 2 | UK | Adult | 18-74 | Both | >1 type | 150 |
| Thomas^12^ | 2000 | Pefloxacin | Placebo | 3 | France | Adult | NR | HSCT | >1 type | 162 |
| Carlson^13^ | 1997 | Ciprofloxacin | No antibiotic | 2 | US | Adult | NR | Cancer | Solid tumor | 90 |
| Horiike^14^ | 1995 | Norfloxacin | Non-absorbable | 2 | Japan | Adult | 34-82 | Cancer | Hematologic malignancy | 23 |
| Brodsky^15^ | 1993 | Ciprofloxacin or Norfloxacin | No antibiotic | 2 | Argentina | Adult | 17-54 | Cancer | Hematologic malignancy | NR |
| Maiche^16^ | 1993 | Ciprofloxacin or Ofloxacin | No antibiotic | 2 | Finland | Adult | 17-83 | Cancer | >1 type | 59 |
| Talbot^17^ | 1993 | Enoxacin | Placebo | 2 | US | Adult | NR | Cancer | Hematologic malignancy | 119 |
| Tanaka^18^ | 1993 | Tosufloxacin | No antibiotic | 3 | Japan | Adult | 17-80 | Cancer | >1 type | NR |
| Yamada^19^ | 1993 | Norfloxacin | No antibiotic | 2 | Japan | Adult | NR | Cancer | Hematologic malignancy | 111 |
| Martino^20^ | 1992 | Ofloxacin | No antibiotic | 2 | Italy | NR | NR | Cancer | Hematologic malignancy | 60 |
| Sampi^21^ | 1992 | Nofloxacin | No antibiotic | 2 |  | Adult | 16-68 | Both | >1 type | NR |
| Schroeder^22^ | 1992 | Ofloxacin | Placebo | 2 | Germany | Adult | 16-75 | Cancer | Solid tumor | 80 |
| Tsutani^23^ | 1992 | Ofloxacin | No antibiotic | 2 | Japan | Adult | 17-72 | Cancer | Hematologic malignancy | NR |
| Archimbaud^24^ | 1991 | Pefloxacin | Non-absorbable | 2 | France | Adult | NR | Both | >1 type | 150 |
| Lew^25^ | 1991 | Ciprofloxacin | Placebo | 2 | US | Adult | 26-54 | HSCT | >1 type | 26 |
| Moriuchi^26^ | 1990 | Ciprofloxacin | Non-absorbable | 2 | Japan | Adult | 19-68 | Cancer | Hematologic malignancy | NR |
| Winston^27^ | 1990 | Ofloxacin | Non-absorbable | 2 | US | Adult | 18-72 | Cancer | Hematologic malignancy | NR |
| Rafecas^28^ | 1989 | Ciprofloxacin | Placebo | 2 | Spain | Adult | 18-69 | Cancer | Hematologic malignancy | 40 |
| Karp^29^ | 1987 | Norfloxacin | Placebo | 2 | US | Adult | 19-74 | Cancer | Hematologic malignancy | 68 |
| **Fluoroquinolone vs. Trimethoprim-sulfamethoxazole** | | | | | | | | | | |
| Lew^30^ | 1995 | Ciprofloxacin | TMP-SMX | 2 | US | Adult | 21-56 | HSCT | >1 type | 167 |
| Donnelly^31^ | 1992 | Ciprofloxacin | TMP-SMX | 2 | Netherlands | Adult | NR | Cancer | Hematologic malignancy | 278 |
| Mocikova^32^ | 1992 | Ofloxacin | TMP-SMX | 2 | Slovakia | NR | 6-52 | Cancer | Hematologic malignancy | 42 |
| Kern^33^ | 1991 | Ofloxacin | TMP-SMX | 2 | Germany | Adult | 16-68 | Cancer | Hematologic malignancy | 160 |
| Arning^34^ | 1990 | Ciprofloxacin | TMP-SMX | 3 | Germany | Adult | 21-74 | Cancer | Hematologic malignancy | 65 |
| Liang^35^ | 1990 | Ofloxacin | TMP-SMX | 2 | Japan | Both | 12-78 | Cancer | >1 type | 110 |
| Orlandi^36^ | 1990 | Norfloxacin | TMP-SMX | 2 | Italy | Both | 13-69 | Cancer | Hematologic malignancy | 60 |
| Bartoloni^37^ | 1989 | Ofloxacin | TMP-SMX | 2 | Italy | Adult | 20-66 | Cancer | Hematologic malignancy | 19 |
| Cruciani^38^ | 1989 | Norfloxacin | TMP-SMX | 2 | Italy | Child | NR | Cancer | >1 type | 49 |
| Bow^39^ | 1988 | Norfloxacin | TMP-SMX | 2 | Canada | Adult | 17-78 | Cancer | Hematologic malignancy | 75 |
| Dekker^40^ | 1987 | Ciprofloxacin | TMP-SMX | 2 | Netherlands | Adult | 16-79 | Cancer | Hematologic malignancy | 60 |
| **Trimethoprim-sulfamethoxazole vs. No Antibiotic** | | | | | | | | | | |
| Oken^41^ | 1996 | TMP-SMX | No antibiotic | 2 | US | Adult | NR | Cancer | Hematologic malignancy | 57 |
| Ward^42^ | 1993 | TMP-SMX | Placebo | 2 | US | Adult | NR | Cancer | Hematologic malignancy | 51 |
| Priesler^43^ | 1987 | TMP-SMX | No antibiotic | 2 | US | NR | NR | Cancer | Hematologic malignancy | 697 |
| Van Eys^44^ | 1987 | TMP-SMX | No antibiotic | 2 | US | Child | NR | Cancer | Hematologic malignancy | 126 |
| Goorin^45^ | 1985 | TMP-SMX | Placebo | 2 | US | Child | 1-16 | Cancer | Hematologic malignancy | 61 |
| Kovatch^46^ | 1985 | TMP-SMX | Placebo | 2 | US | Child | 3-17 | Cancer | >1 type | 91 |
| Estey^47^ | 1984 | TMP-SMX | No antibiotic | 2 | US | Adult | 16-78 | Cancer | Hematologic malignancy | NR |
| Henry^48^ | 1984 | TMP-SMX | No antibiotic | 2 | US | Adult | 16-69 | Cancer | Hematologic malignancy | 43 |
| Lange^49^ | 1984 | TMP-SMX | No antibiotic | 2 | US | Child | 0.5-16 | Cancer | Hematologic malignancy | 67 |
| Martino^50^ | 1984 | TMP-SMX | No antibiotic | 2 | Italy | Both | NR | Cancer | Hematologic malignancy | NR |
| Zinner^51^ | 1984 | TMP-SMX | Placebo | 2 | Europe | Both | NR | Both | >1 type | 545 |
| Gualtieri^52^ | 1983 | TMP-SMX | Placebo | 2 | US | Adult | 15-85 | Cancer | Hematologic malignancy | 66 |
| Kauffman^53^ | 1983 | TMP-SMX | No antibiotic | 2 | US | Adult | NR | Cancer | >1 type | 55 |
| Inoue^54^ | 1982 | TMP-SMX | Placebo | 2 | Japan | Child | NR | Cancer | Hematologic malignancy | 102 |
| Scaglione^55^ | 1982 | TMP-SMX | No antibiotic | 2 | Argentina | Both | NR | Cancer | >1 type | NR |
| Dekker^56^ | 1981 | TMP-SMX | No antibiotic | 2 | Netherlands | Adult | NR | Cancer | Hematologic malignancy | 58 |
| Weiser^57^ | 1981 | TMP-SMX | No antibiotic | 2 | US | Adult | 20-66 | Cancer | Hematologic malignancy | NR |
| **Cephalosporin vs. No Antibiotic** | | | | | | | | | | |
| Slavin^58^ | 2007 | Cefepime | No antibiotic | 2 | Australia | Adult | NR | HSCT | >1 type | 153 |
| Pignon^59^ | 1990 | Ceftriaxone | Placebo | 2 | France | NR | NR | Both | >1 type | NR |
| Harousseau^60^ | 1989 | Ceftriaxone | No antibiotic | 2 | France | Both | 1-66 | Both | >1 type | 95 |
| Haroussea^61^ | 1987 | Ceftriaxone | No antibiotic | 2 | France | Both | 4-63 | Both | >1 type | NR |
| **Parenteral Glycopeptide vs. No Antibiotic** | | | | | | | | | | |
| Teinturier^62^ | 1995 | Vancomycin | No antibiotic | 2 | France | Both | NR | HSCT | Allogeneic transplant | 155 |
| Lamy^63^ | 1993 | Vancomycin | No antibiotic | 2 | France | Adult | 19-69 | Both | >1 type | 59 |
| Attal^64^ | 1991 | Vancomycin | No antibiotic | 2 | France | Both | 1-65 | HSCT | >1 type | 60 |
| Maraninchi^65^ | 1988 | Vancomycin | No antibiotic | 2 | France | Both | NR | HSCT | >1 type | 60 |
| **Rifampin and Fluoroquinolone vs. Fluoroquinolone** | | | | | | | | | | |
| Gomez-Martin^66^ | 2000 | Ciprofloxacin and Rifampin | Ciprofloxacin | 2 | Spain | Adult | NR | HSCT | >1 type | 130 |
| Hidalgo^67^ | 1997 | Ciprofloxacin and Rifampin | Ciprofloxacin | 2 | Spain | Adult | NR | HSCT | Autologous transplant | 40 |
| Bow^68^ | 1996 | Ofloxacin and Rifampin | Ofloxacin | 3 | Canada | Adult | NR | Both | >1 type | 127 |
| **Others** | | | | | | | | | | |
| Clemons^69^ | 2018 | Ciprofloxacin | G-CSF | 2 | Canada | Adult | 31-85 | Cancer | Solid tumor | 186 |
| Fanci^70^ | 2016 | Ciprofloxacin and Amoxicillin | Ciprofloxacin | 2 | Italy | Adult | 15-77 | Both | >1 type | 61 |
| Eleutherakis-Papaiakovou^71^ | 2010 | Ciprofloxacin and Vancomycin | No antibiotic | 2 | Greece | NR | NR | HSCT | Autologous transplant | 157 |
| Timmers^72^ | 2007 | Levofloxacin | Ciprofloxacin and Phenethicillin | 2 | Netherlands | Adult | 18-71 | Both | >1 type | 245 |
| Lalami^73^ | 2004 | Ciprofloxacin and Amoxicillin / Clavulanate | No antibiotic | 2 | Belgium/Greece | Adult | 31-79 | Cancer | >1 type | 48 |
| Castagnola^74^ | 2003 | Amoxicillin / Clavulanate | Placebo | 2 | Italy | Child | 0-18 | Cancer | >1 type | 173 |
| Lee^75^ | 2002 | Ciprofloxacin and Roxithromycin | No antibiotic | 2 | Korea | Adult | NR | Cancer | Hematologic malignancy | 95 |
| Tjan-Heijnen^76^ | 2001 | Ciprofloxacin and Roxithromycin | Placebo | 2 | Europe | Adult | 33-70 | Cancer | Solid tumor | 163 |
| Beelen^77^ | 1999 | Ciprofloxacin and Metronidazole | Ciprofloxacin | 2 | Germany | Adult | 15-57 | HSCT | Allogeneic transplant | 134 |
| Schroder^78^ | 1999 | Ciprofloxacin and Amphotericin | G-CSF | 2 | Netherlands | Adult | 28-51 | Cancer | Solid tumor | 40 |
| Nagatomo^79^ | 1998 | Norfloxacin and TMP-SMX | TMP-SMX | 2 | Japan | Adult | 43-82 | Cancer | Solid tumor | NR |
| Murase^80^ | 1995 | Ciprofloxacin and TMP-SMX | TMP-SMX | 2 | Japan | Adult | 15-84 | Cancer | >1 type | NR |
| Avril^81^ | 1994 | Ceftazidime and Teicoplanin | No antibiotic | 2 | France | Child | 2-16 | HSCT | >1 type | 60 |
| Baki^82^ | 1994 | Ofloxacin | Tetracycline | 2 | Hungary | Adult | NR | Cancer | Solid tumor | 200 |
| Broun^83^ | 1994 | Norfloxacin and [Penicillin or Vancomycin] | Norfloxacin | 2 | US | Adult | 20-52 | HSCT | Autologous transplant | 43 |
| D'Antonio^84^ | 1994 | Ciprofloxacin | Pefloxacin | 3 | Italy | Adult | 19-73 | Cancer | Hematologic malignancy | 189 |
| Jansen^85^ | 1994 | Ciprofloxacin | Nalidixic acid and Non-absorbable | 2 | US | Adult | 18-77 | Both | >1 type | 105 |
| Kern^86^ | 1994 | Ofloxacin and Roxithromycin | Ofloxacin | 2 | Germany | Adult | 17-77 | Both | >1 type | 141 |
| Zinner^87^ | 1994 | Pefloxacin and Penicillin V | Pefloxacin and Placebo | 2 | Belgium | Adult | 16-80 | Both | >1 type | 551 |
| Arico^88^ | 1992 | TMP-SMX | TMP-SMX less than daily | 2 | Italy | Child | NR | Cancer | Hematologic malignancy | 77 |
| D'Antonio^89^ | 1992 | Norfloxacin | Pefloxacin | 2 | Italy | Adult | 18-75 | Cancer | Hematologic malignancy | 136 |
| Guiot^90^ | 1992 | TMP-SMX | Benzylpenicillin | 2 | Netherlands | Adult | 15-62 | Cancer | >1 type | NR |
| D'Antonio^91^ | 1991 | Ofloxacin | Norfloxacin | 2 | Italy | Adult | 18-74 | Both | >1 type | NR |
| Del Favero^92^ | 1991 | Ciprofloxacin | Norfloxacin | 2 | Italy | Adult | 14-79 | Both | >1 type | 801 |
| Gluckman^93^ | 1991 | Ofloxacin and Amoxicillin | Non-absorbable | 2 | France | Both | 11-54 | HSCT | Allogeneic transplant | 44 |
| Smith^94^ | 1990 | Gentamicin, Piperacillin and Teicoplanin | Gentamicin, Piperacillin and Flucloxacillin | 2 | UK | Adult | 16-68 | Both | >1 type | NR |
| Sampi^95^ | 1989 | Pipemidic acid and Non-absorbable | No antibiotic | 2 | Japan | Adult | 15-63 | Cancer | Hematologic malignancy | NR |
| Gluckman^96^ | 1988 | Pefloxacin and Penicillin | Non-absorbable | 2 | France | Both | 6-38 | HSCT | Allogeneic transplant | 65 |
| Maschmeyer^97^ | 1988 | Ciprofloxacin | Norfloxacin | 4 | Netherlands | Adult | NR | Cancer | Hematologic malignancy | 51 |
| Petersen^98^ | 1988 | Tobramycin, Vancomycin and Ticarcillin OR Cefotaxime and Mezlocillin | No antibiotic | 4 | US | Both | 1-58 | HSCT | Allogeneic transplant | NR |
| Bow^99^ | 1987 | Nalidixic acid | TMP-SMX | 2 | Canada | Adult | NR | Cancer | >1 type | 90 |
| Hughes^100^ | 1987 | TMP-SMX | TMP-SMX less than daily | 2 | US | NR | NR | Cancer | Hematologic malignancy | 167 |
| Rossi^101^ | 1987 | TMP-SMX | TMP-SMX less than daily | 2 | Italy | Child | 0.9-15 | Cancer | Hematologic malignancy | 97 |
| Kurrle^102^ | 1986 | TMP-SMX | Non-absorbable | 2 | Netherlands | Adult | 15-60 | Cancer | Hematologic malignancy | 155 |
| Petersen^103^ | 1986 | Tobramycin, Vancomycin and Ticarcillin | Granulocyte infusion | 2 | US | Both | 1-47 | HSCT | Allogeneic transplant | 149 |
| Petersen^104^ | 1986 | Tobramycin, Vancomycin and Ticarcillin | No antibiotic | 2 | US | Both | 3-49 | HSCT | Allogeneic transplant | 135 |
| Bow^105^ | 1984 | TMP-SMX | TMP | 2 | Canada | Both | NR | Cancer | >1 type | 75 |
| Kramer^106^ | 1984 | TMP-SMX and Erythromycin | Placebo | 2 | US | Adult | 16-70 | Both | >1 type |  |
| Guiot^107^ | 1983 | Nalidixic acid and Non-absorbable | Placebo | 2 | Netherlands | Adult | 18-71 | Cancer | Hematologic malignancy | 42 |
| Kurrle^108^ | 1983 | TMP-SMX | Nalidixic acid and Non-absorbable | 2 | Germany | Adult | 15-69 | Cancer | Hematologic malignancy | 100 |
| Pizzo^109^ | 1983 | TMP-SMX and Erythromycin | Placebo | 2 | US | Both | 1-43 | Cancer | >1 type | 150 |
| Wade^110^ | 1983 | TMP-SMX | Nalidixic acid | 2 | US | Both | 10-72 | Cancer | Hematologic malignancy | 62 |
| Watson^111^ | 1982 | TMP-SMX | Non-absorbable | 2 | UK | NR |  | Both | >1 type | 100 |
| Malarme^112^ | 1981 | TMP-SMX and Non-absorbable | Non-absorbable | 3 | Belgium | Adult | NR | Cancer | >1 type | NR |
| Wade^113^ | 1981 | TMP-SMX | Non-absorbable | 2 | US | Adult | 19-69 | Cancer | Hematologic malignancy | 53 |

*For trials with 3 or more arms, the control group referenced was used in the traditional meta-analyses

Abbreviations: Pub – published; No. – number; US – United States; UK United Kingdom; NR – not reported; TMP-SMX – trimethoprim-sulfamethoxazole; HSCT – hematopoietic stem cell transplantation; G-CSF – granulocyte- colony stimulating factor

**Supplemental Appendix 3: Funnel Plot for Comparison of Fluoroquinolone vs. No Antibiotic or Non-absorbable Antibiotic for the Outcome of Bacteremia**


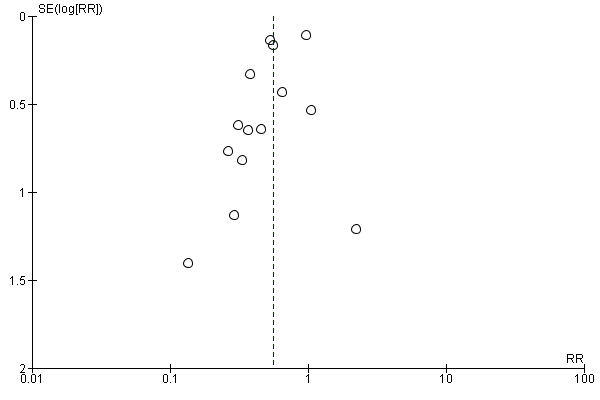


**Legend**: Plot shows estimate of prophylaxis efficacy on the X-axis (risk ratio) where estimates < 1 suggest prophylaxis is better than control. The Y-axis shows the standard error of the log (risk ratio). Asymmetry with missing studies in the right lower quadrant suggest publication bias.

**Supplemental Appendix 4: Funnel Plot for Comparison of Fluoroquinolone vs. No Antibiotic or Non-absorbable Antibiotic for the Outcomes of Overall Mortality**


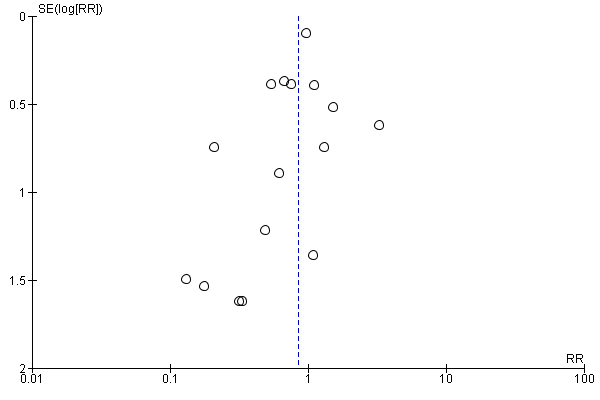


**Legend**: Plot shows estimate of prophylaxis efficacy on the X-axis (risk ratio) where estimates < 1 suggest prophylaxis is better than control. The Y-axis shows the standard error of the log (risk ratio). Asymmetry with missing studies in the right lower quadrant suggest publication bias.

**Supplemental Appendix 5: Network Geometry to Compare Fluoroquinolone vs. Cephalosporin to Prevent Bacteremia**


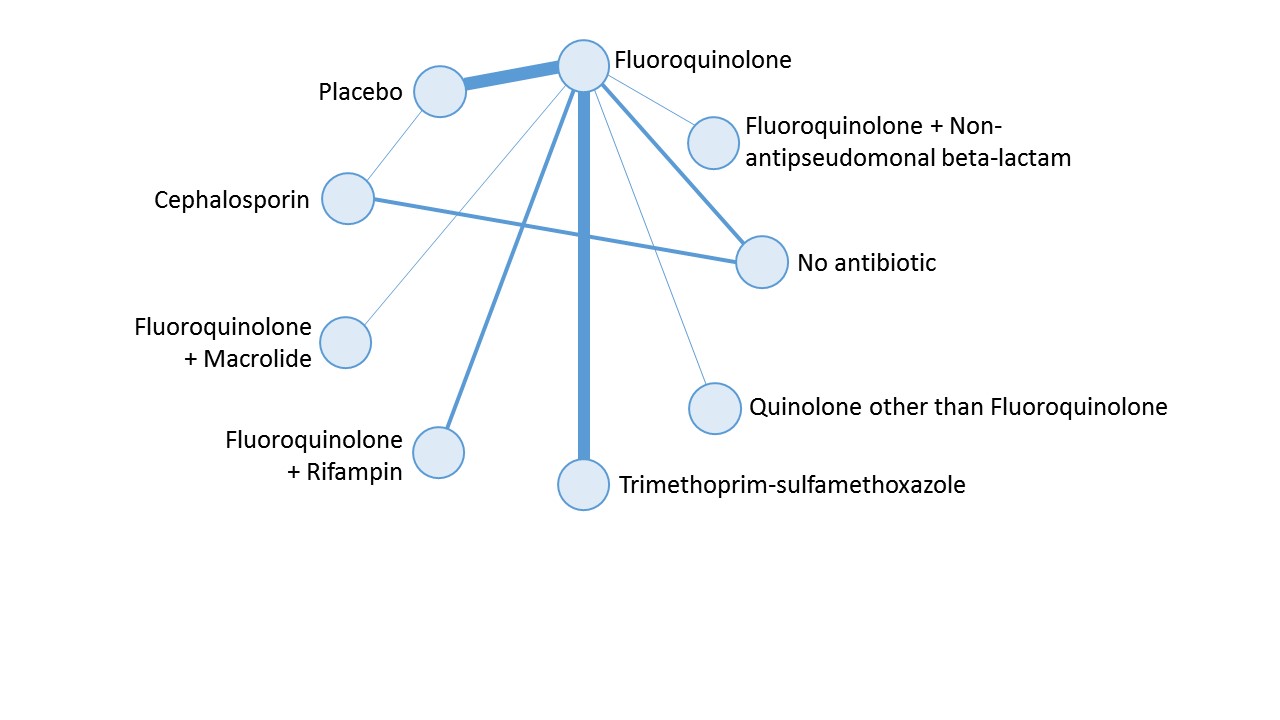


**Legend**: The figure shows the direct and indirect comparisons of studies evaluating the primary outcome of bacteremia. The thickness of the line reflect the number of studies available for a specific comparison. Closed loops such as triangles illustrate where indirect comparisons are available.

**Supplemental Appendix 6: Network Geometry to Compare Levofloxacin vs. Ciprofloxacin to Prevent Bacteremia**


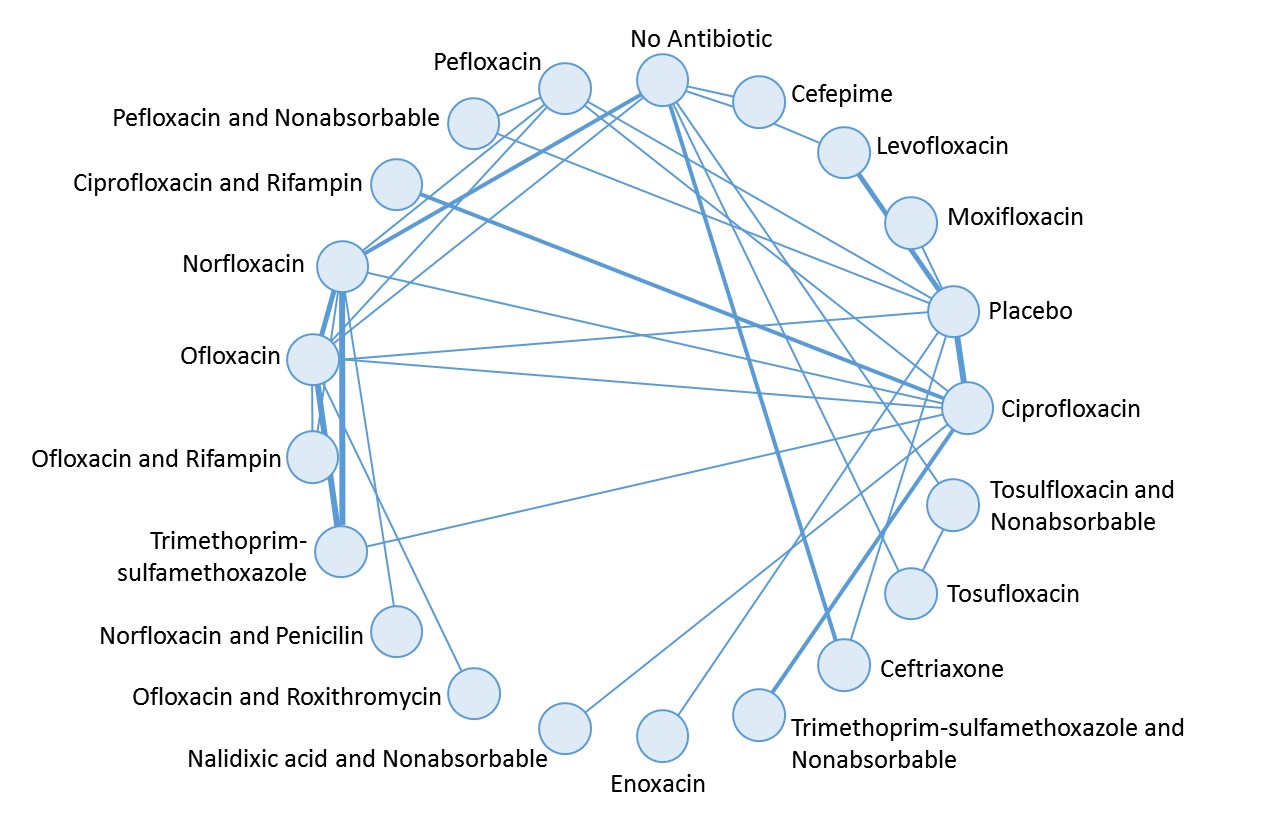


**Legend**: The figure shows the direct and indirect comparisons of studies evaluating the primary outcome of bacteremia. The thickness of the line reflect the number of studies available for a specific comparison. Closed loops such as triangles illustrate where indirect comparisons are available.

**References:**

1. Alexander S, Fisher BT, Gaur AH, et al: Effect of Levofloxacin Prophylaxis on Bacteremia in Children With Acute Leukemia or Undergoing Hematopoietic Stem Cell Transplantation: A Randomized Clinical Trial. JAMA 320:995-1004, 2018

2. Laoprasopwattana K, Khwanna T, Suwankeeree P, et al: Ciprofloxacin reduces occurrence of fever in children with acute leukemia who develop neutropenia during chemotherapy. Pediatric Infectious Disease Journal 32:e94-8, 2013

3. Widjajanto PH, Sumadiono S, Cloos J, et al: Randomized double blind trial of ciprofloxacin prophylaxis during induction treatment in childhood acute lymphoblastic leukemia in the WK-ALL protocol in Indonesia. Journal of Blood Medicine 4:1-9, 2013

4. Vehreschild JJ, Moritz G, Vehreschild MJ, et al: Efficacy and safety of moxifloxacin as antibacterial prophylaxis for patients receiving autologous haematopoietic stem cell transplantation: a randomised trial. International Journal of Antimicrobial Agents 39:130-4, 2012

5. Vesole DH, Oken MM, Heckler C, et al: Oral antibiotic prophylaxis of early infection in multiple myeloma: a URCC/ECOG randomized phase III study. Leukemia 26:2517-20, 2012

6. Schuette W, Nagel S, von Weikersthal LF, et al: Randomized phase III trial of docetaxel plus carboplatin with or without levofloxacin prophylaxis in elderly patients with advanced non-small cell lung cancer: the APRONTA trial. Journal of Thoracic Oncology: Official Publication of the International Association for the Study of Lung Cancer 6:2090-6, 2011

7. Rahman MM, Khan MA: Levofloxacin prophylaxis to prevent bacterial infection in chemotherapy-induced neutropenia in acute leukemia. Bangladesh Medical Research Council Bulletin 35:91-4, 2009

8. Bucaneve G, Micozzi A, Menichetti F, et al: Levofloxacin to prevent bacterial infection in patients with cancer and neutropenia. New England Journal of Medicine 353:977-87, 2005

9. Cullen M, Steven N, Billingham L, et al: Antibacterial prophylaxis after chemotherapy for solid tumors and lymphomas. New England Journal of Medicine 353:988-98, 2005

10. Nenova IS, Ananostev NH, Goranov SE, et al: Fluoroquinolone prophylaxis for bacterial infections in neutropenic patients with hematologic malignancies. Folia Medica (Plovdiv) 43:40-5, 2001

11. Prentice HG, Hann IM, Nazareth B, et al: Oral ciprofloxacin plus colistin: prophylaxis against bacterial infection in neutropenic patients. A strategy for the prevention of emergence of antimicrobial resistance.[Erratum appears in Br J Haematol 2002 Jun;117(4):1002]. British Journal of Haematology 115:46-52, 2001

12. Thomas X, Troncy J, Belhabri A, et al: [Effectiveness of combined vancomycin and pefloxacine in gastrointestinal decontamination for preventing infections after chemotherapy-induced bone marrow aplasia. A randomized double-blind study]. Presse Medicale 29:1745-51, 2000

13. Carlson JW, Fowler JM, Mitchell SK, et al: Chemoprophylaxis with ciprofloxacin in ovarian cancer patients receiving paclitaxel: a randomized trial. Gynecologic Oncology 65:325-9, 1997

14. Horiike S, Ueda Y, Kaneko H, et al: Comparison of norfloxacin with polymyxin for the prevention of infections in granulocytopenic patients with acute leukemia. [Japanese]. Japanese Journal of Chemotherapy 43:207-212, 1995

15. Brodsky AL, Minissale CJ, Melero MJ, et al: [Prophylaxis with fluoroquinolones in patients with neutropenia]. Medicina 53:401-7, 1993

16. Maiche AG, Muhonen T: Granulocyte colony-stimulating factor (G-CSF) with or without a quinolone in the prevention of infection in cancer patients. European Journal of Cancer 29A:1403-5, 1993

17. Talbot GH, Cassileth PA, Paradiso L, et al: Oral enoxacin for infection prevention in adults with acute nonlymphocytic leukemia. The Enoxacin Prophylaxis Study Group. Antimicrobial Agents & Chemotherapy 37:474-82, 1993

18. Tanaka K, Jojima H, Osabe S, et al: Tosufloxacin tosilate for prevention of infection in neutropenic patients with hematological malignancies. A comparison with polymyxin B. [Japanese]. Chemotherapy 41:1183-1190, 1993

19. Yamada T, Dan K, Nomura T: Prevention of bacterial and fungal infections in acute leukemia patients: a new and potent combination of oral norfloxacin and amphotericin B. Internal Medicine 32:710-5, 1993

20. Martino B, Brugiatelli M, Iacopino P, et al: Antibacterial prophylaxis in granulocytopenic patients with systemic blood disease. Randomized study in 60 patients. [Italian]. Haematologica 77:27-28, 1992

21. Sampi K, Maseki N, Hattori M: [A comparison of nystatin with norfloxacin for prevention of infection after consolidation therapy in patients with acute leukemia or autologous bone marrow transplantation: a randomized study]. Gan to Kagaku Ryoho [Japanese Journal of Cancer & Chemotherapy] 19:823-6, 1992

22. Schroeder M, Schadeck-Gressel C, Selbach J, et al: Antibiotic prophylaxis with gyrase inhibitors during cytostatically induced granulocytopenias in patients with solid tumors: A double-blind prospective randomized study. Onkologie 15:476-479, 1992

23. Tsutani H, Imamura S, Ueda T, et al: Prophylactic use of ofloxacin in granulocytopenic patients with hematological malignancies during post-remission chemotherapy. Internal Medicine 31:319-24, 1992

24. Archimbaud E, Guyotat D, Maupas J, et al: Pefloxacin and vancomycin vs. gentamicin, colistin sulphate and vancomycin for prevention of infections in granulocytopenic patients: a randomised double-blind study. European Journal of Cancer 27:174-8, 1991

25. Lew MA, Kehoe K, Ritz J, et al: Prophylaxis of bacterial infections with ciprofloxacin in patients undergoing bone marrow transplantation. Transplantation 51:630-6, 1991

26. Moriuchi Y, Kamihira S, Yamamura M, et al: [Comparison of ciprofloxacin with polymyxin B for infection prophylaxis in neutropenic patients with acute non-lymphocytic leukemia]. Rinsho Ketsueki - Japanese Journal of Clinical Hematology 31:1664-9, 1990

27. Winston DJ, Ho WG, Bruckner DA, et al: Ofloxacin versus vancomycin/polymyxin for prevention of infections in granulocytopenic patients. American Journal of Medicine 88:36-42, 1990

28. Rafecas FJ, Gil E, Martin G, et al: Oral ciprofloxacin in the prophylaxis of bacterial infection in neutropenic patients. A randomized, double-blind, comparative clinical study. [Spanish]. Revista Espanola de Quimioterapia 2:174-177, 1989

29. Karp JE, Merz WG, Hendricksen C, et al: Oral norfloxacin for prevention of gram-negative bacterial infections in patients with acute leukemia and granulocytopenia. A randomized, double-blind, placebo-controlled trial. Annals of Internal Medicine 106:1-7, 1987

30. Lew MA, Kehoe K, Ritz J, et al: Ciprofloxacin versus trimethoprim/sulfamethoxazole for prophylaxis of bacterial infections in bone marrow transplant recipients: a randomized, controlled trial. Journal of Clinical Oncology 13:239-50, 1995

31. Donnelly JP, Maschmeyer G, Daenen S: Selective oral antimicrobial prophylaxis for the prevention of infection in acute leukaemia-ciprofloxacin versus co-trimoxazole plus colistin. The EORTC-Gnotobiotic Project Group. European Journal of Cancer 28A:873-8, 1992

32. Mocikova K, Svac J, Faber E, et al: [Comparison of Tarivid and Biseptol in the prevention of bacterial infections in patients with acute leukemia]. Vnitrni Lekarstvi 38:166-72, 1992

33. Kern W, Kurrle E: Ofloxacin versus trimethoprim-sulfamethoxazole for prevention of infection in patients with acute leukemia and granulocytopenia. Infection 19:73-80, 1991

34. Arning M, Wolf HH, Aul C, et al: Infection prophylaxis in neutropenic patients with acute leukaemia--a randomized, comparative study with ofloxacin, ciprofloxacin and co-trimoxazole/colistin. Journal of Antimicrobial Chemotherapy 26 Suppl D:137-42, 1990

35. Liang RH, Yung RW, Chan TK, et al: Ofloxacin versus co-trimoxazole for prevention of infection in neutropenic patients following cytotoxic chemotherapy. Antimicrobial Agents & Chemotherapy 34:215-8, 1990

36. Orlandi E, Navarra A, Cruciani M, et al: Norfloxacin versus cotrimoxazole for infection prophylaxis in granulocytopenic patients with acute leukemia. A prospective randomized study. Haematologica 75:296-8, 1990

37. Bartoloni A, Fanci R, Orsi A, et al: The influence of ofloxacin versus trimethoprim-sulfamethoxazole on the aerobic flora in granulocytopenic subjects. Journal of Chemotherapy 1:91-4, 1989

38. Cruciani M, Concia E, Navarra A, et al: Prophylactic co-trimoxazole versus norfloxacin in neutropenic children--perspective randomized study. Infection 17:65-9, 1989

39. Bow EJ, Rayner E, Louie TJ: Comparison of norfloxacin with cotrimoxazole for infection prophylaxis in acute leukemia. The trade-off for reduced gram-negative sepsis. American Journal of Medicine 84:847-54, 1988

40. Dekker AW, Rozenberg-Arska M, Verhoef J: Infection prophylaxis in acute leukemia: a comparison of ciprofloxacin with trimethoprim-sulfamethoxazole and colistin. Annals of Internal Medicine 106:7-11, 1987

41. Oken MM, Pomeroy C, Weisdorf D, et al: Prophylactic antibiotics for the prevention of early infection in multiple myeloma. American Journal of Medicine 100:624-8, 1996

42. Ward TT, Thomas RG, Fye CL, et al: Trimethoprim-sulfamethoxazole prophylaxis in granulocytopenic patients with acute leukemia: evaluation of serum antibiotic levels in a randomized, double-blind, placebo-controlled Department of Veterans Affairs Cooperative Study. Clinical Infectious Diseases 17:323-32, 1993

43. Preisler H, Davis RB, Kirshner J, et al: Comparison of three remission induction regimens and two postinduction strategies for the treatment of acute nonlymphocytic leukemia: a cancer and leukemia group B study. Blood 69:1441-9, 1987

44. van Eys J, Berry DM, Crist W, et al: Effect of trimethoprim/sulfamethoxazole prophylaxis on outcome of childhood lymphocytic leukemia. A Pediatric Oncology Group Study. Cancer 59:19-23, 1987

45. Goorin AM, Hershey BJ, Levin MJ, et al: Use of trimethoprim-sulfamethoxazole to prevent bacterial infections in children with acute lymphoblastic leukemia. Pediatric Infectious Disease 4:265-9, 1985

46. Kovatch AL, Wald ER, Albo VC, et al: Oral trimethoprim/sulfamethoxazole for prevention of bacterial infection during the induction phase of cancer chemotherapy in children. Pediatrics 76:754-60, 1985

47. Estey E, Maksymiuk A, Smith T, et al: Infection prophylaxis in acute leukemia. Comparative effectiveness of sulfamethoxazole and trimethoprim, ketoconazole, and a combination of the two. Archives of Internal Medicine 144:1562-8, 1984

48. Henry SA, Armstrong D, Kempin S, et al: Oral trimethoprim/sulfamethoxazole in attempt to prevent infection after induction chemotherapy for acute leukemia. American Journal of Medicine 77:663-6, 1984

49. Lange B, Halpern S, Gale G, et al: Trimethoprim-sulfamethoxazole and nystatin prophylaxis in children with acute lymphoblastic leukemia. European Paediatric Haematology and Oncology 1:231-238, 1984

50. Martino P, Venditti M, Petti MC, et al: Cotrimoxazole prophylaxis in patients with leukemia and prolonged granulocytopenia. American Journal of the Medical Sciences 287:7-9, 1984

51. Zinner SH, Schimpff SC, Klastersky J, et al: Trimethoprim-sulfamethoxazole in the prevention of infection in neutropenic patients. Journal of Infectious Diseases 150:372-9, 1984

52. Gualtieri RJ, Donowitz GR, Kaiser DL, et al: Double-blind randomized study of prophylactic trimethoprim/sulfamethoxazole in granulocytopenic patients with hematologic malignancies. American Journal of Medicine 74:934-40, 1983

53. Kauffman CA, Liepman MK, Bergman AG, et al: Trimethoprim/sulfamethoxazole prophylaxis in neutropenic patients. Reduction of infections and effect on bacterial and fungal flora. American Journal of Medicine 74:599-607, 1983

54. Inoue M, Arai S, Kamiya H, et al: [The prophylactic effect of trimethoprim-sulfamethoxazole against infection among children with acute leukemia]. Rinsho Ketsueki - Japanese Journal of Clinical Hematology 24:26-33, 1983

55. Scaglione C, Tormena AM, Pavlovsky S: [Prophylactic controlled trials with cotrimoxazole in afebrile neutropenic patients with malignant hemopathies]. Sangre 27:912-8, 1982

56. Dekker AW, Rozenberg-Arska M, Sixma JJ, et al: Prevention of infection by trimethoprim-sulfamethoxazole plus amphotericin B in patients with acute nonlymphocytic leukaemia. Annals of Internal Medicine 95:555-9, 1981

57. Weiser B, Lange M, Fialk MA, et al: Prophylactic trimethoprim-sulfamethoxazole during consolidation chemotherapy for acute leukemia: a controlled trial. Annals of Internal Medicine 95:436-8, 1981

58. Slavin MA, Grigg AP, Schwarer AP, et al: A randomized comparison of empiric or pre-emptive antibiotic therapy after hematopoietic stem cell transplantation. Bone Marrow Transplantation 40:157-63, 2007

59. Pignon B, Thiriet L, Aubert D, et al: Evaluation of the efficacy of prophylactic intravenous antibiotherapy with ceftriaxone in post-chemotherapy agranulocytic patients. Nouvelle Revue Francaise d Hematologie 32:249-52, 1990

60. Harousseau J, Milpied N, Reynaud A: Antibiotic prophylaxis with ceftriaxone for severe and durable neutropenia, Medecine et maladies infectieuses, 1989, pp 91-95

61. Harousseau JL, Milpied N, Reynaud AE, et al: [Preventive systemic antibiotherapy with ceftriaxone alone in neutropenic patients treated in a protected environment]. Presse Medicale 16:1737-40, 1987

62. Teinturier C, Hartmann O, Lemerle J, et al: Prevention of gram-positive infections in patients treated with high-dose chemotherapy and bone marrow transplantation: a randomized controlled trial of vancomycin. Pediatric Hematology & Oncology 12:73-7, 1995

63. Lamy T, Michelet C, Dauriac C, et al: Benefit of prophylaxis by intravenous systemic vancomycin in granulocytopenic patients: a prospective, randomized trial among 59 patients. Acta Haematologica 90:109-13, 1993

64. Attal M, Schlaifer D, Rubie H, et al: Prevention of gram-positive infections after bone marrow transplantation by systemic vancomycin: a prospective, randomized trial. Journal of Clinical Oncology 9:865-70, 1991

65. Maraninchi D, Hartman O, Benhamou E, et al: [Prophylaxis of Gram-positive infections after bone marrow graft. Controlled study of the 6-day administration of vancomycin. Intermediate analysis of 60 patients]. Pathologie Biologie 36:915-9, 1988

66. Gomez-Martin C, Sola C, Hornedo J, et al: Rifampin does not improve the efficacy of quinolone antibacterial prophylaxis in neutropenic cancer patients: results of a randomized clinical trial. Journal of Clinical Oncology 18:2126-34, 2000

67. Hidalgo M, Hornedo J, Lumbreras C, et al: Lack of ability of ciprofloxacin-rifampin prophylaxis to decrease infection-related morbidity in neutropenic patients given cytotoxic therapy and peripheral blood stem cell transplants. Antimicrobial Agents & Chemotherapy 41:1175-7, 1997

68. Bow EJ, Mandell LA, Louie TJ, et al: Quinolone-based antibacterial chemoprophylaxis in neutropenic patients: effect of augmented gram-positive activity on infectious morbidity. National Cancer Institute of Canada Clinical Trials Group. Annals of Internal Medicine 125:183-90, 1996

69. Clemons M, Mazzarello S, Hilton J, et al: Feasibility of using a pragmatic trials model to compare two primary febrile neutropenia prophylaxis regimens (ciprofloxacin versus G-CSF) in patients receiving docetaxel-cyclophosphamide chemotherapy for breast cancer (REaCT-TC). Supportive Care in Cancer 11:11, 2018

70. Fanci R, Leoni F, Bosi A, et al: Chemoprophylaxis of bacterial infections in granulocytopenic patients with ciprofloxacin vs ciprofloxacin plus amoxicillin. Journal of Chemotherapy 5:119-23, 1993

71. Eleutherakis-Papaiakovou E, Kostis E, Migkou M, et al: Prophylactic antibiotics for the prevention of neutropenic fever in patients undergoing autologous stem-cell transplantation: results of a single institution, randomized phase 2 trial. American Journal of Hematology 85:863-7, 2010

72. Timmers GJ, Simoons-Smit AM, Leidekker ME, et al: Levofloxacin vs. ciprofloxacin plus phenethicillin for the prevention of bacterial infections in patients with haematological malignancies. Clinical Microbiology & Infection 13:497-503, 2007

73. Lalami Y, Paesmans M, Aoun M, et al: A prospective randomised evaluation of G-CSF or G-CSF plus oral antibiotics in chemotherapy-treated patients at high risk of developing febrile neutropenia. Supportive Care in Cancer 12:725-30, 2004

74. Castagnola E, Boni L, Giacchino M, et al: A multicenter, randomized, double blind placebo-controlled trial of amoxicillin/clavulanate for the prophylaxis of fever and infection in neutropenic children with cancer. Pediatric Infectious Disease Journal 22:359-65, 2003

75. Lee DG, Choi SM, Choi JH, et al: Selective bowel decontamination for the prevention of infection in acute myelogenous leukemia: a prospective randomized trial. Korean Journal of Internal Medicine 17:38-44, 2002

76. Tjan-Heijnen VC, Postmus PE, Ardizzoni A, et al: Reduction of chemotherapy-induced febrile leucopenia by prophylactic use of ciprofloxacin and roxithromycin in small-cell lung cancer patients: an EORTC double-blind placebo-controlled phase III study. Annals of Oncology 12:1359-68, 2001

77. Beelen D, Elmaagacli A, Müller K, et al: Influence of intestinal bacterial decontamination using metronidazole and ciprofloxacin or ciprofloxacin alone on the development of acute graft-versus-host disease after marrow transplantation in patients with hematologic malignancies: final results and long-term follow-up of an open-label prospective randomized trial, Blood, 1999, pp 3267-3275

78. Schroder CP, de Vries EG, Mulder NH, et al: Prevention of febrile leucopenia after chemotherapy in high-risk breast cancer patients: no significant difference between granulocyte-colony stimulating growth factor or ciprofloxacin plus amphotericin B. Journal of Antimicrobial Chemotherapy 43:741-3, 1999

79. Nagatomo A, Watanabe K, Kunikane H, et al: A randomized controlled trial of sulfamethoxazole/trimethoprim plus norfloxacin versus sulfamethoxazole/trimethoprim alone for the prophylaxis of bacteria infection during chemotherapy for lung cancer. Lung Cancer 19:121-5, 1998

80. Murase T: [Chemoprophylaxis of bacterial infections in granulocytopenic patients with new quinolone: a comparison of trimethoprim-sulfamethoxazole (ST) alone with ST plus ciprofloxacin]. Kansenshogaku Zasshi - Journal of the Japanese Association for Infectious Diseases 69:28-32, 1995

81. Avril M, Hartmann O, Valteau-Couanet D, et al: Antiinfective prophylaxis with ceftazidime and teicoplanin in children undergoing high-dose chemotherapy and bone marrow transplantation. Pediatric Hematology & Oncology 11:63-73, 1994

82. Baki M, Bodrogi I, Horti J, et al: [Review of drugs used in the neutropenic period following cytostatic therapy and comparative study of doxycycline and ofloxacin in the treatment of patients with testicular cancer]. Orvosi Hetilap 135:2815-9, 1994

83. Broun ER, Wheat JL, Kneebone PH, et al: Randomized trial of the addition of gram-positive prophylaxis to standard antimicrobial prophylaxis for patients undergoing autologous bone marrow transplantation. Antimicrobial Agents & Chemotherapy 38:576-9, 1994

84. D'Antonio D, Piccolomini R, Iacone A, et al: Comparison of ciprofloxacin, ofloxacin and pefloxacin for the prevention of the bacterial infection in neutropenic patients with haematological malignancies. Journal of Antimicrobial Chemotherapy 33:837-44, 1994

85. Jansen J, Cromer M, Akard L, et al: Infection prevention in severely myelosuppressed patients: a comparison between ciprofloxacin and a regimen of selective antibiotic modulation of the intestinal flora. American Journal of Medicine 96:335-41, 1994

86. Kern WV, Hay B, Kern P, et al: A randomized trial of roxithromycin in patients with acute leukemia and bone marrow transplant recipients receiving fluoroquinolone prophylaxis. Antimicrobial Agents & Chemotherapy 38:465-72, 1994

87. Zinner S, Calandra T, Meunier F, et al: Reduction of fever and streptococcal bacteremia in granulocytopenic patients with cancer. A trial of oral penicillin V or placebo combined with pefloxacin. JAMA 272:1183-9, 1994

88. Arico M, Molinari E, Bacchella L, et al: Prospective randomized comparison of toxicity of two prophylactic regimens of cotrimoxazole in leukemic children. Pediatric Hematology & Oncology 9:35-40, 1992

89. D'Antonio D, Iacone A, Fioritoni G, et al: Comparison of norfloxacin and pefloxacin in the prophylaxis of bacterial infection in neutropenic cancer patients. Drugs Under Experimental & Clinical Research 18:141-6, 1992

90. Guiot HF, van der Meer JW, van den Broek PJ, et al: Prevention of viridans-group streptococcal septicemia in oncohematologic patients: a controlled comparative study on the effect of penicillin G and cotrimoxazole. Annals of Hematology 64:260-5, 1992

91. D'Antonio D, Iacone A, Fioritoni G, et al: Antibacterial prophylaxis in granulocytopenic patients: A randomized study of ofloxacin versus norfloxacin. Current Therapeutic Research - Clinical and Experimental 50:304-311, 1991

92. Del Favero A, Menichetti F, Martino P, et al: Prevention of bacterial infection in neutropenic patients with hematologic malignancies. A randomized, multicenter trial comparing norfloxacin with ciprofloxacin. Annals of Internal Medicine 115:7-12, 1991

93. Gluckman E, Roudet C, Hirsch I, et al: Prophylaxis of bacterial infections after bone marrow transplantation. A randomized prospective study comparing oral broad-spectrum nonabsorbable antibiotics (vancomycin-tobramycin-colistin) to absorbable antibiotics (ofloxacin-amoxicillin). Chemotherapy 37 Suppl 1:33-8, 1991

94. Smith CL, Milliken S, Powles R, et al: Teicoplanin compared to flucloxacillin for antibiotic treatment of neutropenic patients. British Journal of Haematology 76 Suppl 2:6-9, 1990

95. Sampi K, Sakurai M, Kumai R, et al: Combination of pipemidic acid, colistin sodium methanesulfonate and nystatin may be less effective than nystatin alone for prevention of infection during chemotherapy-induced granulocytopenia in acute leukemia. Medical Oncology & Tumor Pharmacotherapy 6:291-6, 1989

96. Gluckman E, Cavazzana M, Devergie A, et al: [Prevention of bacterial infections after bone marrow graft by broad-spectrum oral antibiotics, absorbable (pefloxacin, penicillin) and non absorbable (cephalosporin, gentamycin, bacitracin)]. Pathologie Biologie 36:902-6, 1988

97. Maschmeyer G, Haralambie E, Gaus W, et al: Ciprofloxacin and norfloxacin for selective decontamination in patients with severe granulocytopenia. Infection 16:98-104, 1988

98. Petersen F, Thornquist M, Buckner C, et al: The effects of infection prevention regimens on early infectious complications in marrow transplant patients: a four arm randomized study. Infection 16:199-208, 1988

99. Bow EJ, Rayner E, Scott BA, et al: Selective gut decontamination with nalidixic acid or trimethoprim-sulfamethoxazole for infection prophylaxis in neutropenic cancer patients: relationship of efficacy to antimicrobial spectrum and timing of administration. Antimicrobial Agents & Chemotherapy 31:551-7, 1987

100. Hughes WT, Rivera GK, Schell MJ, et al: Successful intermittent chemoprophylaxis for Pneumocystis carinii pneumonitis. New England Journal of Medicine 316:1627-32, 1987

101. Rossi MR, Banfi P, Cappuccilli M, et al: Prospective randomized comparison of two prophylactic regimens with trimethoprim-sulfamethoxazole in leukemic children: a two year study. European Journal of Cancer & Clinical Oncology 23:1679-82, 1987

102. Kurrle E, Dekker AW, Gaus W, et al: Prevention of infection in acute leukemia: a prospective randomized study on the efficacy of two different drug regimens for antimicrobial prophylaxis. Infection 14:226-32, 1986

103. Petersen FB, Buckner CD, Clift RA, et al: Prevention of nosocomial infections in marrow transplant patients: a prospective randomized comparison of systemic antibiotics versus granulocyte transfusions. Infection Control 7:586-92, 1986

104. Petersen FB, Buckner CD, Clift RA, et al: Laminar air flow isolation and decontamination: a prospective randomized study of the effects of prophylactic systemic antibiotics in bone marrow transplant patients. Infection 14:115-21, 1986

105. Bow EJ, Louie TJ, Riben PD, et al: Randomized controlled trial comparing trimethoprim/sulfamethoxazole and trimethoprim for infection prophylaxis in hospitalized granulocytopenic patients. American Journal of Medicine 76:223-33, 1984

106. Kramer BS, Carr DJ, Rand KH, et al: Prophylaxis of fever and infection in adult cancer patients. A placebo-controlled trial of oral trimethoprim-sulfamethoxazole plus erythromycin. Cancer 53:329-35, 1984

107. Guiot H, Broek P, Meer J, et al: Selective antimicrobial modulation of the intestinal flora of patients with acute nonlymphocytic leukemia: a double-blind, placebo-controlled study, Journal of infectious diseases, 1983, pp 615-623

108. Kurrle E, Bhaduri S, Krieger D, et al: Antimicrobial prophylaxis in acute leukaemia: prospective randomized study comparing two methods of selective decontamination. Klinische Wochenschrift 61:691-8, 1983

109. Pizzo PA, Robichaud KJ, Edwards BK, et al: Oral antibiotic prophylaxis in patients with cancer: a double-blind randomized placebo-controlled trial. Journal of Pediatrics 102:125-33, 1983

110. Wade JC, de Jongh CA, Newman KA, et al: Selective antimicrobial modulation as prophylaxis against infection during granulocytopenia: trimethoprim-sulfamethoxazole vs. nalidixic acid. Journal of Infectious Diseases 147:624-34, 1983

111. Watson JG, Powles RL, Jameson B: Co-trimoxazole versus non-absorbable antibiotics in acute leukemia. Lancet 1:6-9, 1982

112. Malarme M, Meunier-Carpentier F, Klastersky J: Vancomycin plus gentamicin and cotrimoxazole for prevention of infections in neutropenic cancer patients (a comparative, placebo-controlled pilot study). European Journal of Cancer & Clinical Oncology 17:1315-22, 1981

113. Wade JC, Schimpff SC, Hargadon MT, et al: A comparison of trimethoprim-sulfamethoxazole plus nystatin with gentamicin plus nystatin in the prevention of infections in acute leukemia. New England Journal of Medicine 304:1057-62, 1981
